# Integrative analysis of genetic data sets reveals a shared innate immune component in autism spectrum disorder and its co-morbidities

**DOI:** 10.1186/s13059-016-1084-z

**Published:** 2016-11-14

**Authors:** Sumaiya Nazeen, Nathan P. Palmer, Bonnie Berger, Isaac S. Kohane

**Affiliations:** 1 Computer Science and Artificial Intelligence Laboratory, Massachusetts Institute of Technology, 77 Massachusetts Avenue, Cambridge, 02139 MA USA; 2 Department of Biomedical Informatics, Harvard Medical School, 25 Shattuck Street, Boston, 02115 MA USA; 3 Department of Mathematics, Massachusetts Institute of Technology, 77 Massachusetts Avenue, Cambridge, 02139 MA USA

**Keywords:** Autism spectrum disorder, Co-morbidities of ASD, Innate immunity pathways, Three-tiered meta-analysis, Gene expression

## Abstract

**Background:**

Autism spectrum disorder (ASD) is a common neurodevelopmental disorder that tends to co-occur with other diseases, including asthma, inflammatory bowel disease, infections, cerebral palsy, dilated cardiomyopathy, muscular dystrophy, and schizophrenia. However, the molecular basis of this co-occurrence, and whether it is due to a shared component that influences both pathophysiology and environmental triggering of illness, has not been elucidated. To address this, we deploy a three-tiered transcriptomic meta-analysis that functions at the gene, pathway, and disease levels across ASD and its co-morbidities.

**Results:**

Our analysis reveals a novel shared innate immune component between ASD and all but three of its co-morbidities that were examined. In particular, we find that the Toll-like receptor signaling and the chemokine signaling pathways, which are key pathways in the innate immune response, have the highest shared statistical significance. Moreover, the disease genes that overlap these two innate immunity pathways can be used to classify the cases of ASD and its co-morbidities vs. controls with at least 70 % accuracy.

**Conclusions:**

This finding suggests that a neuropsychiatric condition and the majority of its non-brain-related co-morbidities share a dysregulated signal that serves as not only a common genetic basis for the diseases but also as a link to environmental triggers. It also raises the possibility that treatment and/or prophylaxis used for disorders of innate immunity may be successfully used for ASD patients with immune-related phenotypes.

**Electronic supplementary material:**

The online version of this article (doi:10.1186/s13059-016-1084-z) contains supplementary material, which is available to authorized users.

## Background

While at an organismal level, two or more diseases may appear unrelated, at the molecular level, it is unlikely that they arise entirely independently of one another. Studies of the human interactome—the molecular network of physical interactions (e.g., protein–protein, gene, metabolic, regulatory etc.) between biological entities in cells—demonstrate that gene function and regulation are integrated at the level of an organism. Extensive patterns of shared co-occurrences also evidence molecular commonalities between seemingly disparate conditions [1].

Indeed, different disorders may share molecular components so that perturbations causing disease in one organ system can affect another [2]. Yet, since the phenotypes appear so different, medical sub-disciplines address the conditions with sometimes wildly differing treatment protocols. If investigators can uncover the molecular links between seemingly dissimilar conditions, the connections may help explain why certain groups of diseases arise together and assist clinicians in their decision-making about best treatments. Knowledge of shared molecular pathology may also provide therapeutic insights for repositioning of existing drugs [3].

Such thinking has emerged most recently in neuropsychiatry, where many such illnesses do not have clear boundaries in terms of their pathophysiology or diagnosis [4, 5]. Indeed, there is now growing evidence that rare variants ranging from chromosomal abnormalities and copy number variation (CNV) to single nucleotide variation have implications for autism spectrum disorder (ASD) and other neuropsychiatric conditions [6–13]. For example, single nucleotide polymorphisms (SNPs), which overlap genes in common molecular pathways, such as calcium channel signaling, are shared in ASD, attention deficit-hyperactivity disorder, bipolar disorder, major depressive disorder, and schizophrenia [14]. CNVs, especially the rare ones, can explain a portion of the risk for multiple psychiatric disorders [10, 13]. For example, the 16*p*11.2 CNV encompassing around 600 kb (chr 16:29.5, 30.2 Mb) has been implicated in multiple psychiatric disorders with the deletions being associated with ASD, developmental delay, and intellectual disability, and duplications being associated with ASD, schizophrenia, bipolar disorder, and intellectual disability [10, 13, 15–19]. However, pathogenic variations are observed in only about 30 % of the ASD-affected individuals [12, 20–23] and these variations often fail to explain the idiopathic (non-syndromic) ASD cases as well as why ASD-affected individuals suffer from many other non-neuropsychiatric conditions.

To complement the evidence of genome-wide pleiotropy across neuropsychiatric diseases, rather than looking at one neurodevelopmental disease (ASD) and comparing it to other seemingly, brain-related diseases, we expand our exploration outside of the brain to conditions related to other organ systems that co-occur with ASD. Recent studies based on electronic health records [24, 25] have identified various co-morbidities in ASD, including seizures [26, 27], gastrointestinal disorders [28, 29], ear infections and auditory disorders, developmental disorders, sleep disorders [30], muscular dystrophy [31–33], cardiac disorders, and psychiatric illness [34, 35].

In this paper, we introduce an integrative gene expression analysis to identify a shared pathophysiological component between ASD and 11 other diseases, namely, asthma, bacterial and viral infection, chronic kidney disease, cerebral palsy, dilated cardiomyopathy, ear infection, epilepsy, inflammatory bowel disease (IBD), muscular dystrophy, schizophrenia, and upper respiratory infection, that have at least 5 % prevalence in ASD patients [24, 25]. We asked the question, “Do these disease states—which are not included in the definition of ASD but co-occur at a significantly high frequency—illuminate dysregulated pathways that are important in ASD?” We reasoned that such pathways may offer previously hidden clues to shared molecular pathology.

Other investigators have integrated genomic data from genome-wide association studies and non-synonymous SNP studies for multiple immune-related diseases, revealing that combining genetic results better identified shared molecular commonalities [36]. We believe that adopting an integrative approach not only at the gene level but also at the biochemical pathway and disease levels will power the results still further.

Here we describe results from a novel three-tiered meta-analysis approach to determine molecular similarities between ASD and 11 of its co-morbid conditions. For every disease condition, we (i) looked for statistically significant differentially expressed genes, (ii) identified their enrichment in canonical pathways, and (iii) determined the statistical significance of the shared pathways across multiple conditions. We are unaware of any analyses that go from population-based co-morbidity clusters of ASD to a multi-level molecular analysis at anywhere near this breadth.

Our results unearth several innate-immunity-related pathways—specifically, the Toll-like receptor and chemokine signaling pathways—as significant players in ASD and all but three of its examined co-morbidities. Candidate genes in these two pathways significantly overlap in conditions of ASD, asthma, bacterial and viral infection, chronic kidney disease, dilated cardiomyopathy, ear infection, IBD, muscular dystrophy, and upper respiratory infection. Candidate genes did not appear to be significantly shared in cerebral palsy, epilepsy, or schizophrenia. Notably, although bacterial and viral infection, respiratory infection, ear infection, IBD, and asthma have well-known connections with the immune system, we demonstrate that innate immunity pathways are shared by ASD and its co-morbidities, irrespective of whether they are immunity-related diseases or not.

Since both Toll-like receptor signaling and chemokine signaling pathways play crucial roles in innate immunity, the results suggest that this first-line defense system (which protects the host from infection by pathogens and environmental triggers) may be involved in ASD and specific co-morbidities. If the profiles of genetic susceptibility pathways in relation to environmental triggers can be ascertained, they may help in defining new treatments, such as vaccination [37] or other tolerization therapies [38]. Those may help individuals and families who are at high risk for ASD to prevent and/or treat immune-related phenotypes of the illness.

## Results

### Three-tiered meta-analysis pipeline

**Fig. 1:**
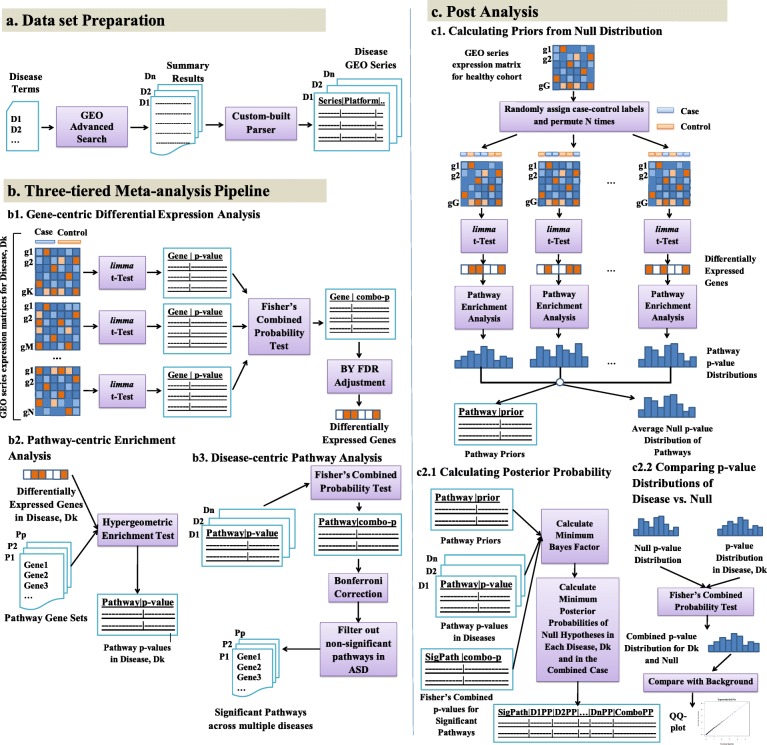
Three-tiered meta-analysis pipeline. **a** Data preparation: Select the GEO series relevant to ASD and co-morbid diseases. **b** Three tiers: (1) For each disease, select significant genes from differential expression analysis of GEO series with a Fisher’s combined test with *p*<0.05 after Benjamini–Yekutieli (BY) FDR adjustment. (2) For each disease, select significant pathways from hypergeometric enrichment analysis with *p*<0.05. (3) Identify significant shared pathways across diseases using Fisher’s combined test with *p*<0.05 after Bonferroni FDR correction. Exclude the non-significant pathways in ASD. **c** Post analysis. (1) Using the gene expression data from a healthy cohort, generate a null distribution of pathway *p* values and calculate prior probabilities of pathways being significant by chance. (2.1) Using the prior probabilities, pathway *p* values in each individual disease, and the Fisher’s combined *p* values of significant pathways across diseases, calculate minimum Bayes factors and minimum posterior probabilities of null hypotheses for each significant pathway in each disease and in the combined case. (2.2) Combine the pathway *p* value distribution of each disease with the average null distribution of *p* values using Fisher’s combined probability test and compare the combined *p* value distribution with the background chi-squared distribution using a QQ plot for significance. Identify the significant pathways using the combined *p* values, minimum posterior probabilities, and QQ plots. *ASD* autism spectrum disorder, *BY* Benjamini–Yekutieli correction, *FDR* false discovery rate, *GEO* Gene Expression Omnibus, *QQ* plot, quantile–quantile plot

We examined ASD and 11 of its most common co-morbidities (Table 1) through a three-tiered lens of gene, pathway, and disease. Figure 1 shows our three-tiered meta-analysis pipeline. Differential analysis of expression data from 53 microarray studies (see Additional file 1: Table S1) related to the 12 disease conditions revealed different numbers of significant genes per disease depending on different false discovery rate (FDR) corrections (shown in Table 2). The complete lists of *p* values per gene per disease under different FDR corrections are given in Additional file 2. To select the most informative FDR correction test, we looked at the accuracy of classification of cases vs. controls for each disease using the disease gene sets selected under different FDR corrections. We found the Benjamini–Yekutieli (BY) adjustment to be the most informative and accurate—classification accuracy being at least 63 % using the genes selected under BY adjustment as features for a support vector machine (SVM) classifier. This was true for all the diseases examined (see “Methods” section as well as Additional file 3: Figure S1 for details).

**Table 1:** Co-morbidities of autism spectrum disorders

Disease group	Clinical manifestations	References
Multisystem disorders (congenital anomalies, auditory disorders,	Asthma	Becker, 2007 [100];
infections, gastro-intestinal disorders, cardiac disorders etc.)		Doshi-Velez, Ge,
		and Kohane, 2014 [25]
	Bacterial and viral infections	Atladóttir et al., 2010 [78];
		Atladóttir et al., 2012 [79];
		Garbett et al., 2012 [63];
		Hagberg, Gressens,
		and Mallard, 2012 [101]
	Chronic kidney disease	Curatolo et al., 2004 [102];
		Loirat et al., 2010 [103]
	Cerebral palsy	Surén et al., 2012 [104];
		Doshi-Velez, Ge,
		and Kohane, 2014 [25]
	Dilated cardiomyopathy	Witchel, Hancox,
		and Nutt, 2003 [105];
		Bilder et al., 2013 [106]
	Ear infection/otitis media	Konstantareas and
		Homatidis, 1987 [107];
		Rosenhall et al., 1999 [108];
		Porges et al., 2013 [109]
	Inflammatory bowel disease	Horvath et al., 1999 [28];
	(Crohn’s disease, ulcerative	Horvath and Perman, 2002 [29];
	colitis)	Walker et al., 2013 [110]
	Muscular dystrophy	Wu et al., 2005 [31];
		Hendriksen and Vles, 2008 [32];
		Hinton et al., 2009 [33];
		Kohane et al., 2012 [24]
	Upper respiratory infection	Shavelle, Strauss,
		and Pickett, 2001 [111];
		Porges et al., 2013 [109];
		Bilder et al., 2013 [106]
Seizures	Epilepsy	Mouridsen et al., 1999 [26];
		Tuchman and Rapin, 2002 [27];
		Surén et al., 2012 [104];
		Bilder et al., 2013 [106]
Psychiatric disorders	Schizophrenia	Morgan, Roy,
		and Chance, 2003 [34];
		Tabarés-Seisdedos
		and Rubenstein, 2009 [112];
		Ingason et al., 2011 [113];
		Smoller et al., 2013 [14];
		Murdoch and State, 2013 [114]

**Table 2:** Number of differentially expressed genes selected under different FDR corrections for different diseases

	Bonferroni	BY	BH	None ^a^
ASD	157	1258	5104	9176
Asthma	238	852	2501	5555
Bacterial and viral infection	1613	3630	6016	8183
Chronic kidney disease	66	416	3771	12577
Cerebral palsy	93	220	646	2352
Dilated cardiomyopathy	146	349	908	3455
Ear infection/otitis media	1629	3867	6708	6708
Epilepsy	5	4	12	2242
Inflammatory bowel disease	831	2547	4771	6897
Muscular dystrophy	207	517	1303	3885
Schizophrenia	54	149	508	2881
Upper respiratory infection	32	59	172	2664

Significance cutoff of *p*<0.05

*ASD* autism spectrum disorder, *BY* Benjamini–Yekutieli, *BH* Benjamini–Hochberg, *FDR* false discovery rate

^a^No FDR correction

Hypergeometric enrichment analysis on individual pathway gene sets from the Kyoto Encyclopedia of Genes and Genomes (KEGG), BioCarta, Reactome, and the Pathway Interaction Database (PID) collections, as well as on the combined gene set of all canonical pathways, helped us to obtain a *p* value per pathway per disease. For different pathway gene set collections, the complete lists of *p* values per pathway in each disease are provided in Additional file 4. Combining the *p* values per pathway across all the diseases using Fisher’s combined probability test [39] and correcting for multiple comparisons using Bonferroni correction, we measured the shared significance of pathways across ASD and its co-morbidities (see “Methods” section for details). After selecting any pathway that had an adjusted *p* value <0.05 as significant and filtering out the pathways that are not significant in ASD, we found a list of pathways that are dysregulated in ASD and at least one of its co-morbidities (see Additional file 4).

**Fig. 2:**
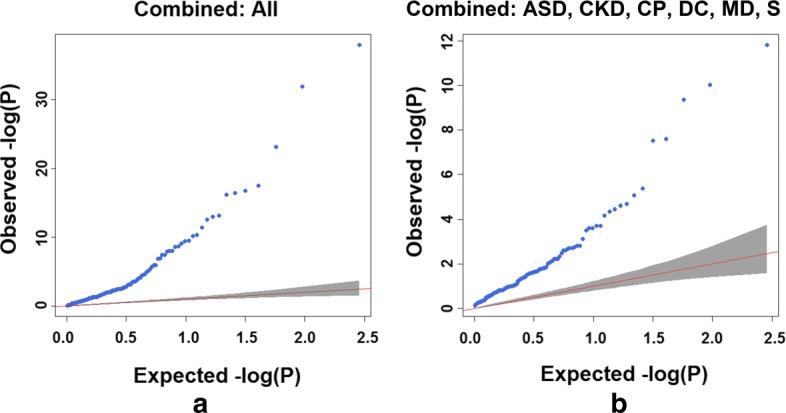
Quantile–quantile plots showing *p* value distributions for a combined analysis. It combines pathway *p* values across **a** ASD and all its co-morbidities, and **b** ASD and its non-immune-related co-morbidities. *ASD* autism spectrum disorder, *CKD* chronic kidney disease, *CP* cerebral palsy, *DC* dilated cardiomyopathy, *MD* muscular dystrophy, *S* schizophrenia

To confirm that the presence of multiple significant pathways among ASD and its co-morbidities was due to shared biology, we estimated minimum Bayes factors (BFs) and minimum posterior probabilities of the null hypothesis for each of the significant KEGG pathways in ASD and its co-morbidities (Fig. 1 and Additional file 5). The priors for the pathways were estimated from 100 null distributions of *p* values generated by differential expression analysis and pathway analysis performed on the gene expression data of a healthy cohort (GEO accession GSE16028) (see Fig. 1 and “Methods” section for details). Looking at the significant pathway *p* values in each disease and their corresponding posterior probabilities of the null hypothesis, we found that, for the significant *p* values (*p*<0.05), the posterior probabilities of the *p* values being significant by chance were always less than 5 *%*. The quantile–quantile (QQ) plot of combined *p* values of pathways across ASD and its co-morbidities shows marked enrichment of significant *p* values indicative of shared disease biology captured by the pathways tested (Fig. 2 a). The QQ plots of hypergeometric *p* values of pathways in ASD and its co-morbid diseases against theoretical quantiles also show significant enrichment (see Additional file 3: Figure S2). For contrast, we combined pathway *p* values from each disease separately with the null *p* value distribution. When the pathway *p* value distribution in a disease is combined with the null *p* value distribution, the QQ plots do not show much deviation from the background distribution (see Additional file 3: Figure S3), indicating both that there is a lack of shared biology (as expected) and that our analysis does not cause systematic inflation.

### Involvement of innate immunity pathways in ASD and its co-morbidities

The results demonstrate that pathways that are dysregulated across ASD and its co-morbidities with the highest statistical significance (i.e., the lowest Bonferroni-corrected combined *p* value) are all related to innate immunity. For the KEGG, BioCarta, and PID gene sets, the Toll-like receptor signaling pathway was found to be the most significant (Additional file 4). For the KEGG database, the top two significant pathways were Toll-like receptor signaling and chemokine signaling (Table 3 and Additional file 4). The top three significant pathways, revealed from the analysis of the Reactome data set, include chemokine receptor signaling, innate immunity, and Toll-like receptor signaling (Additional file 4). When we expanded our aperture of analysis to the gene sets from all canonical pathways, the Toll-like receptor signaling and chemokine signaling pathways were still found to be the most significantly dysregulated in the disease conditions (Additional file 4). Thus, we primarily focused our attention on these two pathways in ASD and its co-morbidities and then, for completeness, extended to other innate immunity KEGG pathways that were found significantly dysregulated (Table 3).

**Table 3:** KEGG pathways significantly shared among ASD and its co-morbidities ^a^

Pathway	ASD	Asthma	Bacterial	Chronic	Cerebral	Dilated	Ear	Epilepsy	Inflammatory	Muscular	Schizo	Upper	Chi-	*p* value from	Bonferroni
			and viral	kidney	palsy	cardio	infection		bowel	dystrophy	phrenia	respiratory	square	chi-square	corrected
			infection	disease		myopathy			disease			infection	value	distribution	*p* value
Toll-like receptor	0.0048*	5.52E-06*	0.0762	0.0114*	0.6550	0.0034*	4.28E-16*	1	5.93E-05*	0.0210*	1	1.14E-10*	189.1151	1.1745E-32*	1.703E-30*
signaling pathway															
Chemokine signaling	0.0145*	0.0003*	0.000051*	0.2197	0.8628	0.0194*	3.21E-10*	1	1.37E-06*	0.5703	1	8.89E-09*	170.8496	7.0449E-26*	1.022E-21*
pathway															
NOD-like receptor	0.0342*	9.02E-05*	0.0136*	0.0019*	0.4760	0.0019*	1.99E-08*	1	0.0036*	0.7335	1	9.04E-05*	116.9434	1.7813E-17*	2.583E-15*
signaling pathway															
Ribosome	6.49E-13*	0.9647	4.84E-10*	0.1720	0.6006	1	0.984089	1	0.9460	0.0026*	1	1	119.0004	3.68E-17*	5.336E-15*
Spliceosome	6.70E-05*	0.9541	6.39E-06*	0.2965	0.3831	0.2746	0.920135	1	1.36E-05*	0.5081	0.1721	1	82.5337	9.9149E-09*	1.438E-06*
Leukocyte trans-	0.0023*	0.8201	0.0110*	0.0797	0.0002*	0.8164	0.097372	1	0.1238	7.63E-06*	0.5000	1	75.6280	9.962E-09*	1.445E-06*
endothelial migration															
Regulation of actin	0.0234*	0.9080	0.2734	0.1131	0.0745	0.0355*	0.227981	1	0.2032	5.90E-05*	0.1330	1	73.9701	2.7324E-05*	0.003962*
cyto-skeleton															
Tight junction	0.0359*	0.5613	0.4111	0.1064	0.0005*	0.8542	0.303934	1	0.1900	0.0006*	1	1	56.2763	6.9114E-05*	0.010022*

*ASD* autism spectrum disorder, *KEGG* Kyoto Encyclopedia of Genes and Genomes

^a^The entries with value ‘1’ indicate where there was no overlap between the pathway and the disease gene set

^*^Entries with significant *p* values

Both Toll-like receptor signaling and chemokine signaling pathways are key pathways in the innate immune response mechanism. Toll-like receptors are the most common pattern recognition receptors that recognize distinct pathogen-associated molecular patterns and participate in the first line of defense against invading pathogens. They also play a significant role in inflammation, immune cell regulation, survival, and proliferation. Toll-like receptors activate various signal transduction pathways, which in turn activate expression and synthesis of chemokines, which together with cytokines, cell adhesion molecules, and immunoreceptors, orchestrate the early host response to infection. At the same time they represent an important link in the adaptive immune response [40]. Our study revealed that the KEGG Toll-like receptor signaling pathway, by itself, was significantly dysregulated (with a combined *p* value of 1.7×10^−30^ after Bonferroni correction) in ASD, asthma, chronic kidney disease, dilated cardiomyopathy, ear infection, IBD, muscular dystrophy, and upper respiratory infection with the minimum posterior probability of appearing significant by chance being at most 1 *%*. In addition, the KEGG chemokine signaling pathway was found significantly dysregulated (with a combined *p* value of 1.02×10^−21^ after Bonferroni correction) in ASD, asthma, bacterial and viral infection, dilated cardiomyopathy, ear infection, IBD, and upper respiratory infection with the minimum posterior probability of appearing significant by chance being at most 2.4 *%* in each case. These findings indicate the role of immune dysfunction in this wide range of seemingly unconnected disease conditions. Although there is some experimental evidence linking an abnormal chemokine response to Toll-like receptor ligands associated with autism [41, 42], no study so far has linked them to the co-morbidities suffered by ASD-affected individuals.

When we looked at the other significant KEGG pathways, we found two others involved in innate immunity, namely, the NOD-like receptor signaling and leukocyte transendothelial migration pathways. The NOD-like receptor signaling pathway, by itself, was significantly dysregulated (with a combined *p* value of 2.6×10^−15^ after Bonferroni correction and a minimum posterior probability of the null hypothesis at most 4 *%*) in ASD, asthma, bacterial and viral infection, chronic kidney disease, dilated cardiomyopathy, ear infection, IBD, and upper respiratory infection. The leukocyte transendothelial migration pathway was significantly dysregulated (with a combined *p* value of 1.4×10^−6^ after Bonferroni correction and a minimum posterior probability of the null hypothesis at most 1.7 *%*) in ASD, asthma, cerebral palsy, and muscular dystrophy. Some NOD-like receptors recognize certain types of bacterial fragments; others induce caspase-1 activation through the assembly of multi-protein complexes called inflammasomes, which are critical for generating mature pro-inflammatory cytokines in concert with the Toll-like receptor signaling pathway. While the Toll-like receptor, chemokine, and NOD-like receptor signaling pathways have more to do with the recognition of infectious pathogens and initiating response, the leukocyte transendothelial migration pathway orchestrates the migration of leukocytes from blood into tissues via a process called diapedesis, which is vital for immune surveillance and inflammation. During this diapedesis of leukocytes, the leukocytes bind to endothelial cell adhesion molecules and then migrate across the vascular endothelium to the site of infection. Notably, increased permeability of the blood–brain barrier favoring leukocyte migration into the brain tissue has been implicated in ASD before [43], but not as a shared transcriptomic commonality among its co-morbidities.

To confirm that the presence of multiple significant innate-immunity-related pathways among ASD and its co-morbidities was due to shared biology, we repeated the combined *p* value analysis excluding the immune-related diseases (bacterial and viral infection, asthma, IBD, upper respiratory infection, and ear infection). Innate immunity pathways (leukocyte transendothelial migration, Toll-like receptor signaling, and NOD-like receptor signaling pathways) still appeared among the most significant dysregulated pathways shared by ASD, cerebral palsy, chronic kidney disease, and muscular dystrophy. The QQ plot of combined *p* values of pathways across ASD and its non-immune-related co-morbidities shows marked enrichment of significant *p* values indicative of the shared disease biology of these conditions (Fig. 2 b). Additional file 1: Table S2 shows the most significant KEGG pathways that are shared by ASD and its non-immune-related co-morbidities. For other pathway gene set collections, the complete lists of Fisher’s combined *p* values per pathway per disease are provided in Additional file 6.

### Disease–innate immunity pathway overlap at gene level

To examine the shared innate immunity KEGG pathways through a finer lens, we examined the genes that overlapped with them (Table 4 and Additional file 3: Figure S4). Although these pathways have a broad involvement in a variety of diseases, a small number of genes in these pathways appear dysregulated most often in ASD and its co-morbidities. Thus, we took a closer look at the genes that are shared by ASD and at least one of its co-morbid conditions.

**Table 4:** Differentially expressed genes in ASD and co-morbidities that overlap with innate immunity pathways

	Toll-like receptor signaling pathway	Chemokine signaling pathway	NOD-like receptor signaling pathway	Leukocyte transendothelial migration pathway
Autism spectrum disorder	TLR9, MAP2K4, CCL4, LY96,	CCL4, JAK2, GRK7, CCL17,	BIRC3, MAPK13, SUGT1, PSTPIP1,	TXK, NCF2, JAM2, GNAI2, GNAI3,
	CD14, TAB2, MAP2K2, MAPK13,	CCL21, CCL22, GNB3, GNAI2,	PYCARD, TAB2, BIRC2	CLDN23, ACTN3, ICAM1, ACTN1, MAPK13,
	MAP2K1, TBK1, TLR1, TLR2	CCR2, CXCR3, GNAI3, CCR10,		CD99, RAP1B, CLDN14, MSN
		ADCY6, PREX1, HCK, MAP2K1,		
		RAP1B		
Asthma	STAT1, IKBKE, NFKB1, RELA,	STAT1, CCL2, GNB4, JAK2,	CXCL1, RIPK2, BIRC3, CCL2,	TXK, ACTN2, ICAM1
	TLR7, TICAM1, IL8, IFNAR1,	CCL20, NFKB1, RELA, CXCL5,	IL8, CASP5, NFKB1, RELA,	
	IFNAR2, TICAM2, CD40, CXCL9,	XCR1, PLCB1, CXCL1, PRKCD,	CXCL2, IL6	
	TLR3, IL6, IRF7	HCK, IL8, CCL1, CXCL2,		
		CXCL9, LYN		
Chronic kidney disease	JUN, CTSK, NFKBIA, FOS	CCL17, NFKBIA, CXCR6	NFKBIA, HSP90AA1, NLRC4,	CLDN16, ACTN4, CLDN9
			HSP90AB1	
Cerebral palsy	CD14	CCL2	CCL2	JAM3, MMP2, VCAM1, ACTN4, ACTG1,
				MSN, CTNNA3
Dilated cardiomyopathy	MYD88, LY96, CD14, NFKBIA,	CCL2, CCL11, CCL8, NFKBIA,	RIPK2, CCL2, CCL11, CCL8,	PIK3R1
	MAP2K1, PIK3R1	MAP2K1, CCR1, PIK3R1	NFKBIA	
Ear infection	JUN, CD86, STAT1, CCL3,	STAT3, STAT1, STAT2, CCL2,	CASP8, CXCL1, RIPK2, TNF,	TXK, NCF4, VCAM1, PIK3R5, CLDN23,
	MYD88, CCL5, CCL4, LBP,	CCL3, CCL5, CCL4, CX3CR1,	BIRC3, CCL2, CCL5, CCL11,	CLDN10, CLDN8, MYL9, CLDN5, ICAM1,
	TLR6, MAP3K8, CD14, IKBKE,	CCL11, CCL7, CXCL14, JAK3,	CCL7, MEFV, CASP1, TNFAIP3,	ACTN4, CLDN19, CLDN22, RASSF5,
	NFKB1, NFKBIA, PIK3R5, TLR5,	JAK2, CCL17, CCL20, CCL19,	MAPK3, NFKB1, NFKBIB, NFKBIA,	CLDN7, CLDN4, PIK3R2
	ITIRAP, RELA, TOLLIP, CXCL11,	CCL22, NFKB1, NFKBIB, NFKBIA,	IL18, RELA, IL1B, NLRP3,	
	TLR7, TLR8, CXCL10, CASP8,	PIK3R5, RELA, GNG7, FGR, GNG11,	BIRC2, CXCL2, IL6	
	TNF, IL12B, MAP2K3, MAP2K1,	GNGT2, XCL1, CXCL5, ADCY3, CXCL11,		
	MAP2K6, MAPK3, IL1B, CD40,	ADCY2, CXCL10, CXCL1, PLCB3,		
	TBK1, CXCL9, TLR3, TLR4,	CXCR5, CXCR2, GNG8, HCK,		
	TLR1, FOS, TLR2, IL6,	MAP2K1, CCR5, CCR7, MAPK3,		
	IRF7, PIK3R2	CXCL16, CCR1, CXCL13, CXCL2,		
		CXCL3, CXCL9, LYN, PIK3R2		
Epilepsy	–	–	–	–
	Toll-like receptor signaling pathway	Chemokine signaling pathway	NOD-like receptor signaling pathway	Leukocyte transendothelial migration pathway
Inflammatory bowel disease	CD86, MYD88, CCL4, LY96,	CCL2, CCL4, CCL11, CCL7,	CHUK, CXCL1, BIRC3, CCL2,	TXK, ITGA4, NCF4, MMP9, NCF2,
	MAP3K8, AKT1, CD14, CTSK,	AKT1, CCL18, ARRB2, CCL20,	CARD6, CCL11, CCL7, IL8,	MYL12A, THY1, GNAI2, MYL5, RHOH,
	SPP1, TOLLIP, CXCL11, TLR8,	GNG5, GNB3, GNB2, CCL24,	CASP5, CASP1, MAPK3, IL1B,	CLDN8, MYL9, CLDN15, MYL12B, RAP1A,
	CXCL10, TICAM1, CHUK, IL8,	PRKX, GNG10, GNAI2, GNG11,	NLRP1, CXCL2, HSP90AB1	PIK3CA, MSN, VAV3
	MAP2K3, IL12A, MAPK3, IRF3,	XCL1, CXCL11, CXCL6, CCR10,		
	IL1B, PIK3CA, CXCL9, TLR4,	CXCL10, ADCY6, CHUK, CXCL1,		
	TLR1, TLR2	PLCB3, CXCR2, CXCR1, HCK,		
		IL8, ADCY4, PRKCZ, MAPK3,		
		CCR1, XCL2, CXCL13, RAP1A,		
		PF4, CXCL2, CXCL3, PF4V1,		
		PIK3CA, CXCL9, PPBP, VAV3,		
		LYN		
Bacterial and viral infection	TLR9, MYD88, CCL5, LY96,	STAT3, STAT2, CCL5, DOCK2,	CHUK, RIPK2, CCL5, NOD1,	TXK, ITGAM, NCF4, MMP9, NCF2, VAV1,
	CD14, NFKBIA, TLR5, TLR8,	JAK2, VAV2, NRAS, NFKBIA,	CARD6, CCL8, CARD8, CASP5,	VASP, MYL12A, VAV2, ITGB2, CTNNA1,
	CHUK, IRAK4, MAP2K7, IRF3,	GNG7, GNG5, GNB2, GNG10,	NOD2, NFKBIA, IKBKG, PYCARD,	GNAI3, EZR, PLCG1, RHOH, PRKCA,
	IKBKG, TICAM2, IL1B, TBK1,	GNG11, ADCY3, CXCR3, CCL4L1,	NLRC4, IL1B, BIRC2	ESAM, RAC2, CD99, ITK, NCF1, CYBA,
	TLR4, TLR1, FOS, TLR2,	GNAI3, GNB1, SOS2, CHUK,		CYBB, MYL5, RHOA
	IRF7	RAF1, RHOA, CXCR2, CXCR1,		
		PRKCD, HCK, RAC2, RASGRP2,		
		ADCY4, CCR3, CCR4, CXCR6,		
		CCR7, GRB2, IKBKG, HRAS,		
		GSK3B, CCR1, ITK, NCF1,		
		PPBP, LYN		
Muscular dystrophy	LY96, CD14, CTSK, SPP1,	GNG10, HCK, MAP2K1, GNG12	PYCARD	JAM3, MMP2, NCF2, VCAM1, ITGB2,
	MAP2K1, FOS			JAM2, MYL5, CD99, ACTG1, MYL12B,
				MSN, CYBA
Schizophrenia	–	–	–	CD99
Upper respiratory infection	CCL4, CXCL11, CXCL10, IFNB1,	STAT2, CCL2, CCL4, CCL7,	CCL2, CCL7, IL6	–
	CXCL9, IL6, IRF7	CXCL11, CXCL10, CXCL9		

*ASD* autism spectrum disorder

**Fig. 3:**
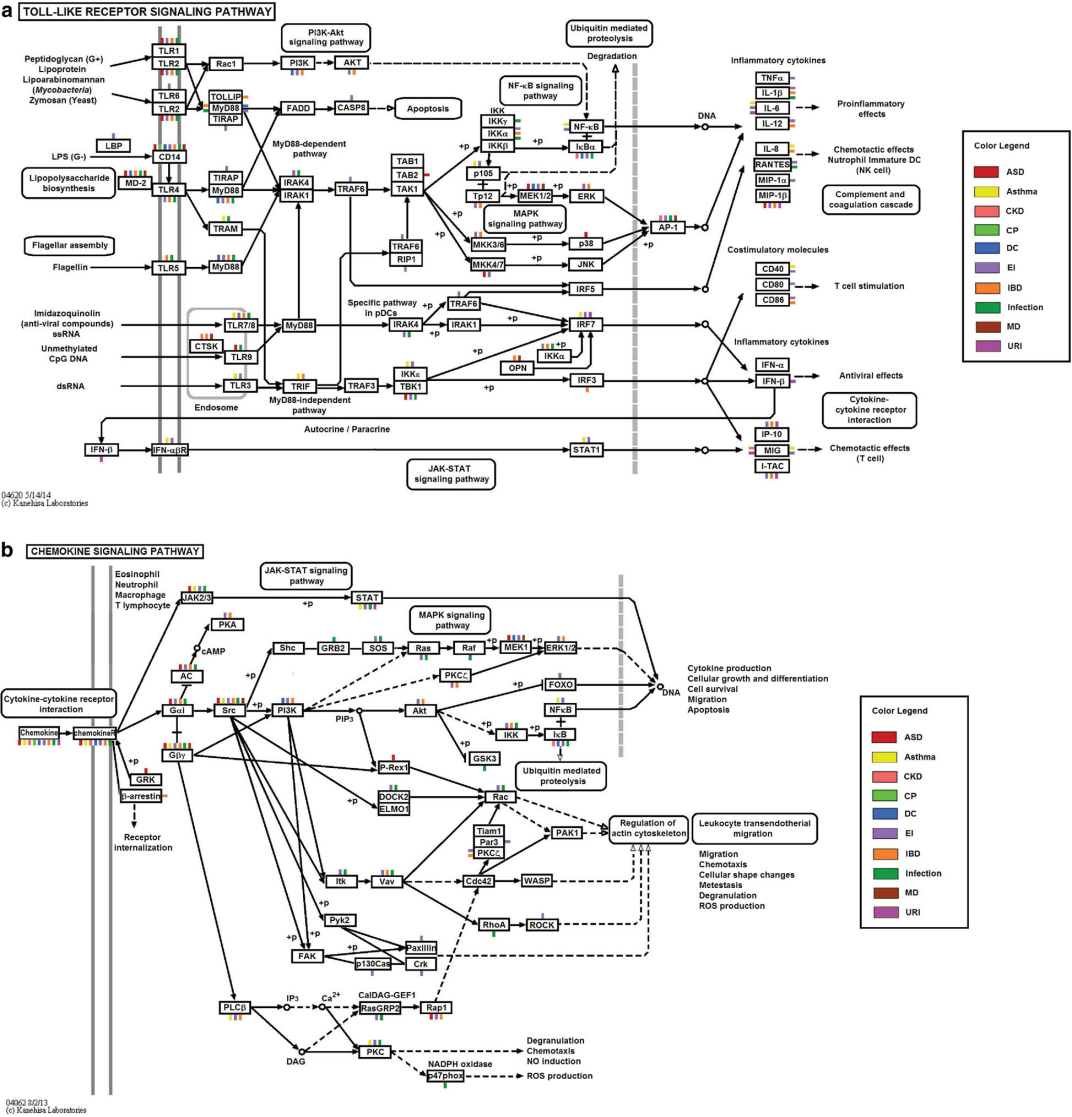
**a** Toll-like receptor signaling pathway color-tagged by co-morbidity findings. **b** Chemokine signaling pathway color-tagged by co-morbidity findings. Genes were mapped onto corresponding KEGG pathway using the “user data mapping tool” from KEGG [91, 92]. Genes are represented by *rectangular boxes*on KEGG pathways. We put color tags on a gene to indicate in which diseases it is differentially expressed. Sometimes a set of genes are mapped onto a single *box*. In that case, the color tags on that *box* represent the union set of all diseases in which those genes are differentially expressed. *ASD* autism spectrum disorder, *CKD* chronic kidney disease, *CP* cerebral palsy, *DC* dilated cardiomyopathy, *EI* ear infection, *IBD* inflammatory bowel disease, *Infection* bacterial and viral infection, *KEGG* Kyoto Encyclopedia of Genes and Genomes, *MD* muscular dystrophy, *URI* upper respiratory infection

In the Toll-like receptor signaling pathway, as shown in Fig. 3 a, commonly shared, differentially expressed genes include CD14 and LY96 (also known as MD-2), responsible for mediating the lipopolysaccharide response, which itself has been shown to create an autism-like phenotype in murine model systems [44], but has never been linked to the shared biology of ASD, cerebral palsy, dilated cardiomyopathy, muscular dystrophy, and IBD. The widely expressed Toll-like receptors, especially, TLR1, TLR2, and TLR9, mediate the recognition of foreign substances, including infectious pathogens, and the regulation of the subsequent cytokine production required for the immune response. Although these genes have been known to be involved in immunity-related conditions, they have not been implicated in the co-occurrence of such conditions in ASD patients. Other genes involved were CCL4, also known as Macrophage inflammatory protein 1 *β* (MIP-1 *β*), which is the most upregulated chemokine in natural killer cells of children with autism [45]; MAPK21, a gene upstream of the MAP-kinases that mediates multiple intra- and extra-cellular signals; JUN (a subunit of transcription factor AP-1), which regulates gene expression in response to a variety of stimuli, including cytokines, growth factors, stress, and bacterial and viral infections; SPP1 (also known as OPN), a cytokine that upregulates expression of interferon- *γ* (IFN- *γ*), which itself has been implicated in ASD and other diseases characterized by social dysfunction [46]; and TBK1, a gene that can mediate NF *κ*B activation in response to certain growth factors and is often considered as a therapeutic target for inflammatory diseases.

In the chemokine pathway, as shown in Fig. 3 b, the commonly shared genes include the chemokines (e.g., CCL4, which had altered expression levels in asthma and ear infection) and MAP-kinases (e.g., MAP2K1, which had altered expression levels in ASD, dilated cardiomyopathy, ear infection, and muscular dystrophy). The HCK gene, which belongs to the Src family of tyrosine kinases, showed altered expression levels in ASD, asthma, IBD, ear infection, bacterial and viral infection, and muscular dystrophy. Considering HCK’s role in microglia and macrophages in controlling proliferation and cell survival [47], this finding is not surprising. JAK2, which is dysregulated in ASD and its multiple immune-related co-morbidities, regulates STAT3 activity, which in turn transduces interleukin-6 (IL-6) signals. Increased IL-6 in the maternal serum has been known to alter fetal brain development, impairing social behaviors in the offspring [48, 49]. The alpha and beta subunits of G-proteins, dysregulated in ASD, asthma, IBD, and bacterial and viral infections, are important signaling molecules, which are often considered to have weak links to a number of brain conditions. The RAP1B gene, a member of the RAS family, regulates multiple cellular processes including cell adhesion, growth and differentiation, and integrin-mediated cell signaling. This protein also plays a role in regulating outside-in signaling in platelets, and G-protein coupled receptor signaling. Thus, it may be of importance.

In the NOD-like receptor signaling pathway, the genes NOD1 and NOD2 drive the activation of NF *κ*B and MAPK, the production of cytokines, and apoptosis. The BIRC2 and BIRC3 genes (which had altered expressions in ASD, asthma, ear infection, and bacterial and viral infections) are members of the inhibitor-of-apoptosis protein family and are key regulators of NOD1 and NOD2 innate immunity signaling. In the leukocyte transendothelial migration pathway, the TXK gene, which is a non-receptor tyrosine kinase (with altered expression in ASD, ear infection, IBD, and bacterial and viral infections), specifically regulates IFN- *γ* gene transcription and the development, function, and differentiation of conventional T cells and nonconventional NKT cells. Mutation of the TXK gene has been identified to be a segregating factor for a number of neurodevelopmental disorders, including ASD, bipolar disorder, and intellectual disabilities [50].

Besides the immune-related ones, Table 3 documents several other pathways and gene sets including the ribosome and spliceosome gene sets, which have roles in genetic information processing and translation and the actin cytoskeleton regulation pathway, which controls various cellular processes like cell motility. Neuronal signal processing and neuron motility have often been associated with ASD, thus these findings are not surprising. The genes in the tight junction pathway mediate cell adhesion and are thought to constitute the intra-membrane and para-cellular diffusion barriers. These findings implicate the involvement of these cellular processes in the shared pathology of ASD and its co-morbidities.

### Discriminatory power of innate immunity pathway genes

**Fig. 4:**
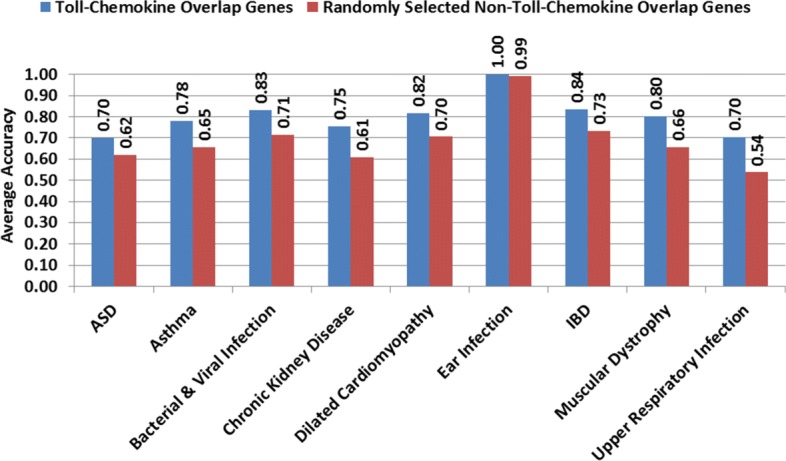
Accuracy of classification for case–control groups in different diseases using differentially expressed genes that overlap in the KEGG Toll-like receptor signaling and chemokine signaling pathways versus randomly selected disease genes that do not overlap in the innate immunity pathways. Diseases for which the differentially expressed genes are not over-represented in the Toll-like receptor signaling and chemokine signaling pathways, are omitted here. *ASD* autism spectrum disorder, *IBD* inflammatory bowel disease, *KEGG* Kyoto Encyclopedia of Genes and Genomes

We assessed the discriminatory power of the innate immunity pathway genes, by taking the union of the genes from the chemokine signaling and Toll-like receptor signaling pathways and performing threefold SVM classification of cases vs. controls for each of the 12 disease conditions. We could achieve an average accuracy of at least 70 % (Fig. 4). We also performed the same classification using the same number of randomly selected genes that do not overlap with these pathways. With randomly selected genes, the classification accuracy was much lower. This result suggests that the genes that have altered expressions in the diseases examined and are present in these innate immunity pathways were sufficient to partially distinguish the disease states from the controls. When we included the overlapping genes in the NOD-like receptor signaling and transendothelial migration pathways in this analysis, the classification accuracy was at least 65 % (see Additional file 3: Figure S5), which was still better than for the randomly selected non-immune genes. In fact, a recent functional genomic study showed that immune/inflammation-related genes can provide reasonable accuracy in the diagnostic classification of male infants and toddlers with ASD [51].

## Discussion

This study bridges previous analyses based on the electronic health records of the co-morbidities of large populations of individuals with ASD and the gene expression profiles of each of these co-morbid diseases as well as ASD against their respective control cases. We have identified that the most significantly and consistently dysregulated pathways shared by these diseases are the innate immunity signaling pathways. For most of these disorders, the genes in these pathways can classify the disorders with respect to their controls with moderate accuracy, further evidence of the extent of the dysregulation in these pathways.

In contrast to traditional approaches that look at a group of disorders of the same organ system, we have focused on ASD and its co-morbidities, which often occur in different organ systems, with a view to finding their shared genetics. It would have been ideal to perform the study on a sufficiently large cohort of ASD patients having enough representatives of all the co-morbid diseases, but in practice, such a study is currently infeasible due to cost constraints and/or patient availability. Thus, to perform this study with existing data sets for ASD and its co-morbidities, we make use of the power of statistics and computation. First, we look at the functional genomic makeup of patients with ASD and its co-morbid diseases separately, and then find the commonalities between them. Some of the microarray studies we looked at have small sample sizes, which gives rise to the possibility of poor random error estimates and inaccurate statistical tests for differential expression. For this reason, we selected limma *t*-statistics, an empirical Bayes method [52], which is reportedly one of the most effective methods for differential expression analysis even for very small data sets [53]. To find the combined significance of the pathways across multiple diseases, we used Fisher’s combined probability test [39], because, it gives a single test of significance for a number of not-so-correlated tests of significance performed on very heterogeneous data sets. When the individual tests do not appear as significant, yet have a combined effect, Fisher’s combined *p* value can indicate whether the probability of the combined effect is on the whole lower than would often have been obtained by chance. Notably, a significant statistic from Fisher’s test implies that the pathway is involved in the biology of at least one of the diseases. Thus, to ensure that the combined significant statistic is due to the shared biology of multiple diseases, we calculate minimum BFs and minimum posterior probabilities of significance by chance for each significant pathway, and also compare the combined *p* value distributions of diseases and the null data set using QQ plots. We draw our conclusions using a combination of the *p* values and the posteriors to avoid any systematic bias inherent to the methods used.

As expected for a neurological disease, the pathways that are most significantly dysregulated in ASD are often the pathways involved in neuronal signaling and development, synapse function, and chromatin regulation [12]. Similarly, for immune-related diseases, like, asthma, IBD, and various infections, the role of innate immunity pathways is well documented in individual studies [54–60]. Despite some controversy, in the last 15 years, experimental evidence has also pointed in the direction of dysregulated immunological signaling in at least some subsets of individuals with autism. This evidence includes findings of an abnormal chemokine response to Toll-like receptor ligands associated with autism in experimental studies [41, 42], and differential gene and protein expression in the central nervous system and peripheral blood of patients with ASD [35, 41, 61–68]. Many reports suggest the alteration of the activation, amount, and distribution of microglia, a representative immune cell in the brain, and its autophagy to be involved in ASD [69–72]. A recent study implicates adaptive immune dysfunction, in particular, disruption of the IFN- *γ* signaling driven anti-pathogen response, to be related to ASD and other diseases characterized by social dysfunction [46]. However, that dysregulation of innate immunity pathways connects ASD with some of its non-immune-related co-morbidities (e.g., chronic kidney disease, cerebral palsy, and muscular dystrophy) is rather intriguing.

That the innate immunity pathways are shared between ASD and the other co-morbid states does not mean that all cases of ASD are characterized by a disorder in these pathways. For example, in our previous work we have shown that although, on average, the gene expression profile of children with ASD shows dysregulated innate immunity signaling, this is a reflection of the smaller number of individuals with ASD who are outliers in this pathway [73]. With our growing understanding of the heterogeneity of ASD and the characterization of ASD populations with distinct co-morbidity associations [25], the integrative analysis we describe here may, therefore, implicate a subset of individuals with ASD with innate immune dysregulation that is either the result of genetic vulnerabilities [74] or particular exogenous stimuli such as infections or disordered microbiome ecologies [75].

Although it is tempting to consider that innate immunity signaling is primarily driven by external environmental stimuli such as infection, we have to recognize that the same signaling mechanisms may be repurposed by different organs for different purposes. For example, 21 % of the genes described in the KEGG long-term potentiation pathway (one of the mechanisms underlying synaptic plasticity) overlap with the genes in the Gene Ontology’s collection of immune genes. It may be, as suggested by large epidemiological studies, that sometimes the disorder is in the signaling system and at other times it is because of an external stimulus. Specifically, nationally scaled studies have demonstrated increased autoimmune disease frequency in the parents of children with ASD [76], increased gestational C-reactive protein in mothers of children with ASD [77], and increased frequency of ASD after pregnancies complicated by infection [78, 79]. Some early studies also suggest the infectious exposure may be directly from the gastrointestinal microbiome [80–84], which also can engage the innate immune system. The success of treatment and/or prophylaxis for disorders of innate immunity in some of the diseases that are co-morbid with ASD raises the possibility that similar treatments may also be successful for subsets of those with ASD.

## Conclusions

Over the years, ASD has baffled researchers not only with its heterogeneity, but also its co-occurrence with a number of seemingly unrelated diseases of different organ systems. In this study, we introduced a three-tier meta-analysis approach to capture the shared genetic signals that form the basis of ASD’s co-occurrence with other diseases. For ASD and 11 of its most frequently occurring co-morbidities, we extracted significant differentially expressed genes, measured their enrichment in canonical pathways, and determined the pathways that are shared by the diseases in question in a statistically rigorous fashion. An analysis of this scale for studying ASD and its co-morbidities is unheard of as per our knowledge. Our results reveal the involvement of two disrupted innate immunity pathways – Toll-like receptor signaling and chemokine signaling – in ASD and several of its co-morbidities irrespective of whether they are immune-related diseases or not. We also showed that the disease genes that overlapped with these pathways could discriminate between patients and controls in each disease with at least 70 % accuracy, further proving their importance. As innate immunity pathways are imperative in orchestrating the first line-of-defense mechanism against infection-causing pathogens and environmental triggers, their involvement in ASD and its co-morbidities can be thought of as the missing genetic link for environmental factors in the pathophysiology of ASD. This mindset also raises the possibility that successful treatments for innate immunity disorders may help ASD patients.

## Methods

### Overview of the three-tiered meta-analysis

To analyze genome-wide expression studies across ASD and 11 of its co-morbidities (Table 1), we introduced a step-wise three-tiered meta-analysis pipeline (Fig. 1). Our meta-analysis started at the gene level, in which we first identified the genes that are differentially expressed among cases and controls for a given disease. We then extended this analysis to the pathway level, where we investigated the pathways that were significantly enriched in candidate genes for a given disease. Finally, we identified the pathways that were significant across multiple diseases by newly combining pathway-level results across diseases and performing a Bayesian posterior probability analysis of null hypotheses for pathways in each disease as well as in the combined case. Details are described below.

#### Gene-centric expression analysis per disease

Using the GEOquery package [85] from Bioconductor in R, we downloaded the gene expression data for each disease in gene matrix transposed (GMT) format from the Gene Expression Omnibus (GEO). The accession identifiers for the disease studies are listed in Additional file 1: Table S1. We removed ‘NA’ values from the data and log-normalized the expression values for subsequent analysis. Then, we performed differential expression analysis on each data set using the limma package [52] from Bioconductor in R, and obtained *p* values for each gene in each experiment.

To determine the degree of correlation between the differential expression analyses of the *p* values of data sets selected under each disease, we calculated the pairwise Pearson correlation coefficient of *p* values (Additional file 1: Table S3). Considering a Pearson correlation coefficient of at least 0.30 with *p*<0.05 as significant, we found that the *p* values are not significantly correlated. This lack of correlation allowed us to use Fisher’s combined probability test to calculate combined *p* values for the genes in each disease condition. We used Fisher’s combined probability test as follows:

$$P\sim\chi^{2}=-2\sum_{i=1}^{k}\ln(p_{i}). $$

Here, *p*_*i*_ is the *p* value of test *i*, *χ* ^2^ is the chi-squared distribution, *k* is the number of tests, and *P* is the adjusted *p* value (*p*<0.05 was considered significant).

#### Selecting the most informative FDR correction test for multiple comparisons

To adjust the combined *p* values, we considered different FDR corrections [i.e., Bonferroni, Benjamini–Yekutieli (BY), and Benjamini–Hochberg (BH)]. We also considered the ‘no correction’ case for completeness. We selected the most informative one, based on the level of accuracy we could achieve in classifying cases of a particular disease, vs. controls, using the genes selected under a specific test with a significance cutoff of *p*<0.05. We tested the accuracy of the case–control classification for each of the 53 disease data sets using four different classification methods, namely, naive Bayes method, Fisher’s linear discriminant analysis, *k* nearest neighbor, and SVM. The set of significant genes selected under different FDR corrections was considered as a feature of the classification methods. We performed threefold cross validation and calculated the average accuracy. We selected the FDR correction test that produced the best average accuracy in each disease. See Additional file 3: Figure S1 and the supplementary text on different classification techniques for microarray gene expression data provided in Additional file 7 for more details.

#### Pathway-centric enrichment analysis per disease

From the disease-level gene-centric expression analysis, we obtained a list of significant genes per disease. For each disease, we then performed a hypergeometric enrichment test for each pathway. This test uses the hypergeometric distribution to calculate the statistical significance of *k* or more significant disease genes, out of *n* total genes, appearing in a specific pathway gene set. It helps identify whether or not the specific disease gene set is over-represented in a certain pathway, by providing a *p* value per pathway per disease.

#### Disease-centric analysis of pathways

Once we obtained the *p* values for the pathways per disease, first we calculated the pairwise Pearson correlation of pathway *p* values across diseases (Additional file 1: Table S4). Since the distributions were not significantly correlated (Pearson correlation coefficient <0.30 with *p* value <0.05), we safely assumed the distributions to be independent. Next, we calculated combined *p* values for each pathway across all the diseases using Fisher’s combined probability test. We corrected for multiple comparisons using Bonferroni correction. We defined a significance threshold of adjusted *p* value <0.05 and called any pathway that passed this threshold, significant. We restricted our results to the pathways that appeared significant in ASD.

#### Calculation of priors, minimum BFs, and minimum posterior probabilities of null hypotheses

To estimate the prior probability of pathways, we selected a publicly available GEO study of 109 gene expression profiles of blood drawn from healthy individuals enrolled at a single site (GEO accession: GSE16028). We assigned case–control labels randomly to the samples and performed differential expression analysis using R package limma. We selected differentially expressed genes using uncorrected *p* values (<0.05), because after BY correction none of the genes remained significant. On the significant gene list, we performed hypergeometric enrichment analysis to obtain a pathway *p* value distribution. We repeated this process 100 times to obtain 100 null *p* value distributions. We calculated the prior for each pathway by looking at how many times the pathway appeared significant (*p* value <0.05) during these 100 runs. We took an average of the 100 distributions to obtain the null *p* value distribution.

The null hypothesis for pathway *p* values is that *p* values are uniformly distributed and the alternative hypothesis is that smaller *p* values are more likely than larger *p* values. Following the approach of Sellke, Bayarri, and Berger [86], we estimated the minimum BFs using the following formula:

$$\text{BF} = \begin{cases} -e p \log(p), & \text{if}\ p<\frac{1}{e}, \\ 1, & \text{otherwise}, \end{cases} $$

where *e* is Euler’s constant.

For calculating minimum BFs for *χ* ^2^-distributed test statistics, we used Johnson’s formula [87]:

$$\text{BF} = \begin{cases} (\frac{v}{x})^{-\frac{v}{2}}\exp(-\frac{x-v}{2}), & \text{for}\ x>v, \\ 1, & \text{otherwise}, \end{cases} $$

where *x* is the chi-square statistic that gave rise to the observed *p* value and *v* is the degrees of freedom.

Following Goodman’s approach [88], we used the prior probability distribution drawn from the null data set and the minimum BF to estimate a lower bound on the posterior probability of the null hypothesis based on Bayes’ theorem as follows:

$${} \text{Minimum Posterior Probability} = \left(\! 1+\left(\frac{\text{BF} \times q}{1-q}\right)^{-1}\right)^{-1} $$

where *q* is the prior probability.

The null distributions and priors for all KEGG pathways and the minimum BFs, and minimum posterior probabilities of null hypotheses for KEGG pathways are given in Additional file 5.

### Measuring the discriminatory power of overlapping innate immunity genes

We performed threefold classification and measured the average accuracy of the case–control classification for each disease with the SVM classifier using the union set of the genes from KEGG Toll-like receptor signaling and chemokine signaling pathways shared across ASD and its co-morbidities to see how well the overlapping genes could distinguish the disease state from controls and compared it with the classification accuracy using randomly selected genes that do not overlap with these two pathways (Fig. 4). We repeated the same test for the overlapping genes in the four innate immunity KEGG pathways and compared the classification accuracy with the discriminatory power of randomly selected non-immunity genes (Additional file 3: Figure S5).

### Data set selection

#### Gene expression data sets

We selected 11 disease conditions that co-occur most commonly in ASD patients. Each of these diseases has at least 5 % prevalence in ASD patients [25]. The prevalence of a co-morbid condition can be defined in two ways: (i) the percentage of ASD patients having a co-morbid disease and (ii) the percentage of patients with a co-morbid disease having ASD [24]. The diseases that satisfy either of these criteria include asthma, bacterial and viral infection, cerebral palsy, chronic kidney disease, dilated cardiomyopathy, ear infection/otitis media, epilepsy, IBD, muscular dystrophy, schizophrenia, and upper respiratory infection. Table 1 shows the disease groups along with the literature references.

To identify publicly available studies relevant to these co-morbidities, we performed an extensive literature search of the GEO of the National Center for Biotechnology Information (NCBI) [89, 90]. Using the advanced search tool provided by GEO, we searched series data sets from studies that performed expression profiling by array on either human or mouse. The search results were parsed using a custom-built parser. It identified 1329 GEO studies for ASD and 11 of its co-morbidities that have been publicly available since 2002. We verified the search results by hand to remove false positives. From the hand-curated results, we retained only those series that corresponded to case–control studies and had complete gene annotations supplied by either NCBI or the submitter. We investigated whether case–control studies had matched controls for the disease cases as well as to reduce noise. We made sure that we had at least 30 samples under each disease. For each selected GEO series, the accession identifier as well as abridged study details including the organism, tissue type, platform, and number of samples is provided in Additional file 1: Table S1. To remove the potential for biases that could arise from using gene expression data sets from different array platforms, tissues, and species, we avoided combining the actual measurements of expression values across platforms, tissues, and diseases. Instead, we performed differential expression analysis on each study separately and then combined the *p* values only.

#### Pathway gene sets

We collected 1320 curated pathway gene sets, including those from the KEGG pathways [91, 92], Reactome pathways [93, 94], BioCarta pathways [95], PID pathways [96], SigmaAldrich gene sets, Signaling Gateway gene sets, Signal Transduction KE gene sets, and SuperArray gene sets from the Molecular Signatures Database (MSigDb) version 4.0 [97]. The gene sets were downloaded in GMT format. Of the available gene sets, we used those that were expert-curated: C2:CP (canonical pathways), C2:CP-BioCarta (BioCarta gene sets), C2:CP-KEGG (KEGG gene sets), C2:CP-Reactome (Reactome gene sets), and PID (Pathway Interaction Database gene sets extracted from C2). From the KEGG collection, we excluded the disease- and drug-related gene sets. After excluding too large (>300 genes) and too small (<10 genes) gene sets, 1261, 146, 211, 629, and 196 gene sets remained in these categories, respectively.

## Additional files

Additional file 1 Supplementary tables. This PDF file contains supplementary Tables S1 through S4. (PDF 218 kb)

Additional file 2 Differential expression analysis of genes. This Excel file contains differential expression analysis *p* values per gene per disease under different FDR corrections [i.e., Bonferroni, Benjamini–Yekutieli (BY), and Benjamini–Hochberg (BH)] as well as ‘no correction’. For presentation purposes, the genes that are not significant even under the ’no correction’ case, have been omitted. (XLS 54 kb)

Additional file 3 Supplementary figures. This PDF file contains supplementary Figures S1 through S5 and their captions. (PDF 8110 kb)

Additional file 4 Pathway enrichment analysis. This Excel file contains hypergeometric test *p* values per pathway per disease for KEGG, BioCarta, Reactome, and PID pathway collections as well as all canonical pathway gene sets collected from MSigDB version 4.0., and Fisher’s combined *p* values for ASD and its co-morbidities. (XLS 1444 kb)

Additional file 5 Posterior probability analysis. This Excel file contains the minimum Bayes factors and minimum posterior probabilities for null hypotheses for the significant KEGG pathways. It also contains the 100 pathway *p* value distributions generated by permuting the null data set. Finally, it has Fisher’s combined *p* values for ASD and its co-morbidities with the null *p* value distribution (one sheet per disease). (XLSX 380 kb)

Additional file 6 Pathway enrichment analysis for non-immune diseases. This Excel file contains hypergeometric test *p* values per pathway per disease for KEGG, BioCarta, Reactome, and PID pathway collections as well as all canonical pathway gene sets collected from MSigDB version 4.0, and Fisher’s combined *p* values for ASD and its co-morbidities excluding the immune-related diseases: bacterial and viral infection, asthma, inflammatory bowel disease, upper respiratory infection, and ear infection. (XLS 673 kb)

Additional file 7 Supplementary text. This PDF file contains the supplementary text describing the four different classification techniques we used for classifying cases vs. controls in microarray gene expression data from ASD and its co-morbidities. (PDF 196 kb)

## Abbreviations

ASD: Autism spectrum disorder
BF: Bayes factor
BH: Benjamini– Hochberg correction
BY: Benjamini– Yekutieli correction
CNV: Copy number variation
FDR: False discovery rate
GEO: Gene Expression Omnibus
GMT: Gene matrix transposed
IBD: Inflammatory bowel disease
IFN: Interferon
IN: interleukin
KEGG: Kyoto Encyclopedia of Genes and Genomes
MSigDB: Molecular Signatures Database
NCBI: National Center for Biotechnology Information
PID: Pathway Interaction Database
QQ plot: Quantile– quantile plot
SNP: Single nucleotide polymorphism
SVM: Support vector machine
