# Supplementary material for: Integrative analysis of genetic data sets reveals a shared innate immune component in autism spectrum disorder and its co-morbidities
Source: Genome Biol. 2016 Nov 14;17:228. doi: 10.1186/s13059-016-1084-z (PMC5108086; doi:10.1186/s13059-016-1084-z)
Supplement: Additional file 1 — Supplementary tables. This PDF file contains supplementary Tables S1 through S4. (PDF 218 kb) [file 13059_2016_1084_MOESM1_ESM.pdf]

Supplementary Tables for

"Integrative analysis of genetic datasets reveals a shared innate immune component in autism spectrum disorder and its co-morbidities"

Nazeen *et al.*

Supplementary Tables Index

Table S1: Selected GEO series for ASD and its co-morbidities.	Page 2
Table S2: KEGG pathways significant in ASD and its non-immune related co-morbidities.	Page 3
Table S3: Pairwise Pearson correlation (with significance) of *p*-values of differential expression analyses performed on selected GEO series for ASD and its comorbidities.	Page 4
Table S4: Pairwise Pearson correlation (with significance) of hypergeometric *p*-values of KEGG pathways in ASD and its comorbidities as well as the null data set.	Page 6

Table S1: Selected GEO series for ASD and its co-morbidities.

Disease	Series Accession ID	Platform	Organism	Tissue Type	Number of Samples
ASD	GSE25507	Affymetrix	*Homo sapiens*	Peripheral blood lymphocytes	146
	GSE7329	Agilent	*Homo sapiens*	Lymphoblastoid cells	30
	GSE28521	Illumina	*Homo sapiens*	Brain tissue	79
	GSE26415	Agilent	*Homo sapiens*	Peripheral blood leucocyte	84
	GSE6575	Affymetrix	*Homo sapiens*	Blood tissue	47[1]
	GSE18123	Affymetrix	*Homo sapiens*	Peripheral blood	285
Asthma	GSE19187	Affymetrix	*Homo sapiens*	Nasal epithelial cells	24[1]
	GSE27011	Affymetrix	*Homo sapiens*	White blood cells	54
	GSE45251	Agilent	*Homo sapiens*	Airway smooth muscle cells	16
	GSE470	Affymetrix	*Homo sapiens*	Epithelial tissue	12
	GSE8190	Agilent	*Homo sapiens*	Airway epithelial cells	250
Bacterial & Viral Infection	GSE40396	Illumina	*Homo sapiens*	Whole blood	65
	GSE42026	Illumina	*Homo sapiens*	Whole blood	92
	GSE47172	Affymetrix	*Homo sapiens*	Whole blood	15
	GSE34205	Affymetrix	*Homo sapiens*	PBMC	101
Chronic Kidney Disease	GSE43484	Affymetrix	*Homo sapiens*	Monocytes	6
	GSE41030	Agilent	*Homo sapiens*	Fibroblasts	6
	GSE38117	Agilent	*Mus musculus*	Renal tissue	6
	GSE48041	Agilent	*Mus musculus*	Kidney tissue	24
	GSE15072	Affymetrix	*Homo sapiens*	Peripheral blood mononuclear cells	29
Cerebral Palsy	GSE16447	Affymetrix	*Homo sapiens*	Fibroblasts	9
	GSE31243	Affymetrix	*Homo sapiens*	Skeletal muscle biopsies	40
Dilated Cardiomyopathy	GSE29819	Affymetrix	*Homo sapiens*	Heart tissue	38
	GSE42955	Affymetrix	*Homo sapiens*	Heart tissue	29
Ear Infection	GSE49128	Affymetrix	*Mus musculus*	Middle ear tissue	17
	GSE49122	Affymetrix	*Mus musculus*	Inner ear tissue	14
Epilepsy	GSE32534	Affymetrix	*Homo sapiens*	FFPE peritumoral neurocortex tissue	10
	GSE6834	Ion channel splice array	*Homo sapiens*	Temporal cortex brain tissue	20[1]
	GSE6614	Affymetrix	*Mus musculus*	Brain tissue	28
	GSE47516	Affymetrix	*Mus musculus*	Cerebellum & granule neurons	21
	GSE20977	Illumina	*Homo sapiens*	CNV	15
	GSE22225	Affymetrix	*Homo sapiens*	Lymphocytes	15
	GSE16969	Affymetrix	*Homo sapiens*	Cortical tubers	10
Inflammatory Bowel Disease	GSE11223	Agilent	*Homo sapiens*	Colon epithelial biopsies	202
	GSE3365	Affymetrix	*Homo sapiens*	PBMC	127
	GSE38713	Affymetrix	*Homo sapiens*	Intestinal mucosa	43
	GSE9452	Affymetrix	*Homo sapiens*	Colonic mucosa	26
Muscular Dystrophy	GSE42806	Affymetrix	*Homo sapiens*	Muscle tissue	12
	GSE36398	Affymetrix	*Homo sapiens*	Muscle tissue	50
	GSE9397	Affymetrix	*Homo sapiens*	Muscle biopsies	20
	GSE6011	Affymetrix	*Homo sapiens*	Muscle biopsies	37
Schizophrenia	GSE53987	Affymetrix	*Homo sapiens*	Prefrontal cortex, Striatum, Hippocampus	103[1]
	GSE27383	Affymetrix	*Homo sapiens*	PBMC	72
	GSE48072	Illumina	*Homo sapiens*	Blood	66
	GSE46509	Affymetrix	*Homo sapiens*	Parvalbumin-immunoreactive neurons	16
	GSE37981	Affymetrix	*Homo sapiens*	Pyramidal cells in superior temporal cortex	18
	GSE21935	Affymetrix	*Homo sapiens*	Post-mortem brain tissue	42
	GSE25673	Affymetrix	*Homo sapiens*	hiPSC-derived neurons	24
	GSE21138	Affymetrix	*Homo sapiens*	Prefrontal cortex	59
	GSE17612	Affymetrix	*Homo sapiens*	Post-mortem brain tissue	51
	GSE12654	Affymetrix	*Homo sapiens*	Prefrontal cortex	28[1]
Upper Respiratory Infection	GSE24132	Affymetrix	*Homo sapiens*	Peripheral and cord blood	12
	GSE35940	Agilent	*Mus musculus*	Lung tissue	43

[1] Samples that are not relevant have been excluded.

Table S2: KEGG pathways significantly shared among ASD and its non-immune related co-morbidities[α].

Pathway	ASD	Chronic Kidney Disease	Cerebral Palsy	Dilated Cardiomyopathy	Epilepsy	Muscular Dystrophy	Schizophrenia	Fisher's Combined p-value	Bonferroni adjusted p-value
KEGG_RIBOSOME	6.49E-13	0.172037	0.600632	1	1	0.002591	1	1.50E-12	2.18012E-10
KEGG_LEUKOCYTE_TRANSENDOTHELIAL_MIGRATION	0.002258	0.079742	0.000231	0.816409	1	7.63E-06	0.499981	2.97E-08	4.3048E-06
KEGG_REGULATION_OF_ACTIN_CYTOSKELETON	0.023391	0.11315	0.074492	0.035518	1	5.90E-05	0.133049	4.22E-06	0.000612218
KEGG_TIGHT_JUNCTION	0.035872	0.106418	0.000501	0.854199	1	0.000562	1	8.77E-06	0.001272115
KEGG_TOLL_LIKE_RECEPTOR_SIGNALING_PATHWAY	0.004832	0.011358	0.655014	0.003408	1	0.021005	1	2.03E-05	0.002940207
KEGG_NOD_LIKE_RECEPTOR_SIGNALING_PATHWAY	0.03415	0.001942	0.47601	0.001888	1	0.733508	1	0.000193	0.027937645
KEGG_OXIDATIVE_PHOSPHORYLATION	0.000406	0.684424	0.755777	1	1	0.002298	1	0.000306	0.044396296

[α] Entries indicating significant *p*-values are colored in red. The entries with value '1' indicate the case where there was no overlap between the pathway and the disease-gene set.

Table S3: Pairwise Pearson correlation (with significance) of *p*-values of differential expression analyses performed on selected GEO series for ASD and its comorbidities. The upper triangle of each table contains the Pearson correlation coefficients and the lower triangle contains the corresponding *p*-values. Cells with *p*-value < 0.05 are colored pink in the lower triangle. Corresponding cells with Pearson correlation coefficients in the upper triangle are colored blue. None but one of the blue cells has a correlation coefficient greater than or equal to 0.3.

(a) ASD

	GSE25507	GSE7329	GSE28521	GSE26415	GSE6575	GSE18123
GSE25507		0.0794	0.0182	-0.0241	0.1541	0.1677
GSE7329	0		0.0249	0.0040	0.0662	0.0425
GSE28521	0.084828	0.018488		-0.0135	0.0365	0.0054
GSE26415	0.012431	0.680063	0.264874		-0.1147	0.1359
GSE6575	0	0	0.000527	7.68E-33		0.1059
GSE18123	0	1.50E-08	0.610237	0	0	

(e) Ear Infection

	GSE49122	GSE49128
GSE49122		0.24266
GSE49128	6.10E-292	

(f) Cerebral Palsy

	GSE16447	GSE31243
GSE16447		0.036765
GSE31243	2.37E-05	

(b) Asthma

	GSE19187	GSE27011	GSE45251	GSE470	GSE8190
GSE19187		0.035452	0.015853	-0.00765	0.003702
GSE27011	4.66E-07		0.015651	-0.01574	-0.01487
GSE45251	0.104889	0.109513		0.017844	0.016564
GSE470	0.484999	0.150816	0.181331		0.014934
GSE8190	0.635232	0.056758	0.074998	0.166517	

(g) IBD

	GSE11223	GSE3365	GSE38713	GSE9452
GSE11223		0.048836	0.106442	0.113977
GSE3365	6.14E-08		0.113617	0.112207
GSE38713	0	0		0.300818
GSE9452	0	0	0	

(c) Bacterial and Viral Infection

	GSE40396	GSE42026	GSE47172	GSE34205
GSE40396		0.295331	0.051173	0.082082
GSE42026	0		0.064674	0.113252
GSE47172	1.22E-08	9.70E-13		0.130602
GSE34205	0	0	0	

(h) Muscular Dystrophy

	GSE42806	GSE36398	GSE9397	GSE6011
GSE42806		4.99E-05	0.061912	0.107029
GSE36398	0.996778		-0.00674	0.057128
GSE9397	5.52E-06	0.462326		0.105613
GSE6011	3.55E-15	4.62E-10	0	

(d) Chronic Kidney Disease

	GSE43484	GSE41030	GSE38117	GSE48041	GSE15072
GSE43484		0.057147	0.232712	-0.34469	0.105452
GSE41030	2.30E-10		-0.50721	-0.16014	0.046865
GSE38117	0.423346	0.044924		0.042126	0.340841
GSE48041	0.208331	0.488038	7.40E-09		0.130358
GSE15072	0	2.02E-07	0.233051	0.643321	

(i) Dilated Cardiomyopathy

	GSE29819	GSE42955
GSE29819		0.045878
GSE42955	2.17E-09	

(j) Epilepsy

	GSE32534	GSE6834	GSE6614	GSE47516	GSE20977	GSE22225	GSE16969
GSE32534		0.207271	0.351927	-0.20701	-0.01422	0.051975	0.10904
GSE6834	0.255009		-0.02817	-0.05239	-0.05092	-0.03661	0.092351
GSE6614	0.353	2.21E-09		0.046069	0.115041	0.094221	0.470471
GSE47516	0.5414	2.20E-16	1.42E-05		0.47973	0.178194	0.366258
GSE20977	0.2103	0.6356	0.7218	0.08258		0.014718	0.008314
GSE22225	5.20E-07	0.7319	0.7595	0.5091	0.05244		0.168657
GSE16969	2.20E-16	0.3866	0.1047	0.1629	0.2733	2.20E-16	

(k) Upper Respiratory Infection

	GSE24132	GSE35940
GSE24132		-0.3601
GSE35940	0.170677	

(l) Schizophrenia

	GSE53987	GSE27383	GSE48072	GSE46509	GSE37981	GSE21935	GSE25673	GSE21138	GSE17612	GSE12654
GSE53987		0.062179	-0.03822	0.063908	0.068824	0.072015	-0.00652	0.182676	0.117167	0.001572
GSE27383	1.90E-09		0.049802	0.049936	0.034112	0.080855	-0.02492	0.048017	0.083542	0.012493
GSE48072	0.22423	0.113166		-0.0485	0.016935	0.007609	-0.03061	-0.05324	-0.05513	-0.0111
GSE46509	1.36E-09	2.21E-06	0.030094		0.257697	0.054245	-0.0366	0.031365	0.085963	0.041056
GSE37981	6.69E-11	0.001228	0.449096	0		0.065238	-0.05012	0.031681	0.093971	0.033255
GSE21935	3.66E-11	1.07E-13	0.815961	1.00E-06	3.99E-09		-0.01371	0.010969	0.157804	0.054345
GSE25673	0.569173	0.029607	0.181661	1.98E-06	7.35E-11	0.253018		0.022986	-0.01571	0.000224
GSE21138	0	0.000361	0.176182	0.022052	0.020748	0.433282	0.119577		0.025817	-0.0017
GSE17612	0	6.66E-16	0.079444	4.44E-16	0	0	0.170259	0.055239		0.008755
GSE12654	0.906541	0.350649	0.697173	0.000118	0.001815	8.96E-05	0.983701	0.921557	0.513086	

Table S4: Pairwise Pearson correlation (with significance) of hypergeometric p-values of KEGG pathways in ASD and its comorbidities as well as the null data set. The upper triangle of each table contains the Pearson correlation coefficients and the lower triangle contains the corresponding *p*-values. Cells with *p*-value < 0.05 are colored pink in the lower triangle. Corresponding cells with Pearson correlation coefficients in the upper triangle are colored blue. None of the blue cells has a correlation coefficient greater than or equal to 0.3; thus the independence assumption for data sets is satisfied. Here, ASD = Autism Spectrum Disorder, CKD = Chronic Kidney Disease, CP = Cerebral Palsy, DC = Dilated Cardiomyopathy, EI = Ear Infection, IBD = Inflammatory Bowel Disease, Infection = Bacterial & Viral Infection, MD = Muscular Dystrophy, and URI = Upper Respiratory Infection.

	asd	asthma	inf	ckd	cp	dc	ei	ibd	md	s	uri	NULL
asd		0.004169	1	0.08294	0.045326	0.076967	0.005257	0.097036	0.004987	0.037704	0.006864	0.009253
asthma	0.950635		0.20903	0.02605	0.011955	0.023419	0.2512	0.178736	0.100256	0.153183	0.138483	0.169088
inf	0	0.009048		0.088518	0.223431	0.042385	0.239231	0.203782	0.004987	0.037704	0.006864	0.118191
ckd	0.160367	0.788995	0.293124		0.282498	0.103413	0.278533	0.246013	0.076323	0.33307	0.069892	0.059191
cp	0.443528	0.908953	0.020702	0.010127		0.147305	0.149028	0.080354	0.099201	0.177432	0.021374	0.231012
dc	0.192773	0.799583	0.61148	0.291467	0.181173		0.150154	0.034153	0.081447	0.315967	0.056339	0.094819
ei	0.929215	0.010875	0.00414	0.000824	0.225161	0.142102		0.118462	0.003749	0.028345	0.032568	0.250611
ibd	0.100285	0.026069	0.002743	0.002857	0.410645	0.682358	0.161793		0.069792	0.081573	0.097878	0.056727
md	0.932847	0.089455	0.932847	0.196532	0.092894	0.168056	0.949489	0.23772		0.158418	0.004895	0.211801
s	0.523932	0.009223	0.523932	6.87E-09	0.002511	4.26E-08	0.949489	0.167396	0.007065		0.03701	0.297515
uri	0.907663	0.018711	0.907663	0.23705	0.717962	0.340737	0.582016	0.097356	0.934079	0.531602		0.067065
NULL	0.916464	0.064863	0.164287	0.573031	0.058042	0.363321	0.002723	0.50714	0.022464	0.000323	0.427778	
