# Supplementary material for: Integrative analysis of genetic data sets reveals a shared innate immune component in autism spectrum disorder and its co-morbidities
Source: Genome Biol. 2016 Nov 14;17:228. doi: 10.1186/s13059-016-1084-z (PMC5108086; doi:10.1186/s13059-016-1084-z)
Supplement: Additional file 3 — Supplementary figures. This PDF file contains supplementary Figures S1 through S5 and their captions. (PDF 8110 kb) [file 13059_2016_1084_MOESM3_ESM.pdf]

Supplementary Figures for

"Integrative analysis of genetic datasets reveals a shared innate immune component in autism spectrum disorder and its co-morbidities"

Nazeen *et al.*

Supplementary Figure Legends

Figure S1: Accuracy of different classification methods for case-control group classification in ASD and its co-morbid diseases using genes selected under different false discovery rate (FDR) corrections as features. (A) Naïve Bayes Classification, (B) Fisher's Linear Discriminant Analysis, (C) k-Nearest Neighbor Classification, and (D) Support Vector Machine.

Figure S2: Quantile-quantile plots comparing *p*-value distributions of KEGG pathways across each of ASD and its co-morbidities with theoretical quantiles. The plots are in *log*-scale. (A) ASD, (B) Asthma, (C) Bacterial and viral infection, (D) Chronic kidney disease, (E) Cerebral Palsy, (F) Dilated Cardiomyopathy, (G) Ear infection, (H) IBD, (I) Muscular Dystrophy, (J) Schizophrenia, and (K) Upper Respiratory Infection.

Figure S3: Quantile-quantile plots showing combined *p*-value distribution of KEGG pathways ASD and all its co-morbidities as well as combined *p*-value distribution of pathways for each disease with the null distribution. The combined *p*-values are compared with theoretic quantiles drawn from appropriate chi-square distributions and the null distribution is compared with theoretical quantiles from standard normal distribution.(A) All diseases combined, (B) Null distribution, (C) ASD and null, (D) Asthma and null, (E) Bacterial and viral infection and null, (F) Chronic kidney disease and null, (G) Cerebral Palsy and null, (H) Dilated Cardiomyopathy and null, (I) Ear infection and null, (J) IBD and null, (K) Muscular Dystrophy and null, (L) Schizophrenia and null, and (M) Upper Respiratory Infection and null.

Figure S4: Binary heatmap showing the overlap between the disease gene sets and the union set of genes in the four significant innate immunity pathways from KEGG. A cell marked "Red" indicates an "overlap" whereas a cell marked "Blue" means "no overlap". For presentation purposes, the genes having no overlap with any of the diseases are omitted. Here, ASD = Autism Spectrum Disorder, CKD = Chronic Kidney Disease, CP = Cerebral Palsy, DC = Dilated Cardiomyopathy, EI = Ear Infection, IBD = Inflammatory Bowel Disease, Infection = Bacterial & Viral Infection, MD = Muscular Dystrophy, and URI = Upper Respiratory Infection.

Figure S5: Accuracy of classification for case-control groups in different disease using differentially expressed genes that overlap in the significant innate immunity related KEGG pathways versus randomly selected disease genes that do not overlap in the innate immunity pathways.

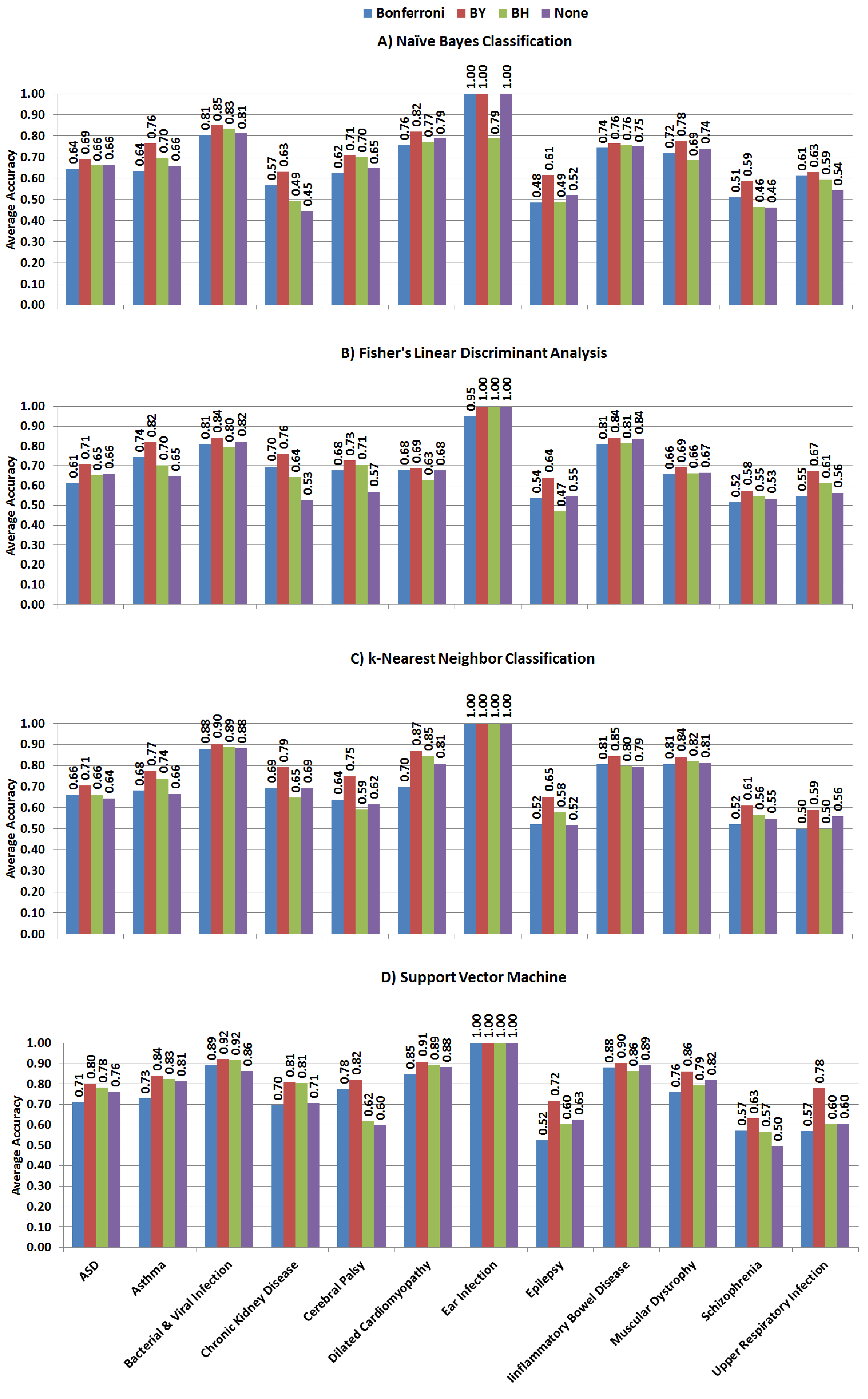

Figure S1: Accuracy of different classification methods for case-control group classification in ASD and its co-morbid diseases using genes selected under different false discovery rate (FDR) corrections as features. (A) Naïve Bayes Classification, (B) Fisher's Linear Discriminant Analysis, (C) *k*-Nearest Neighbor Classification, and (D) Support Vector Machine.

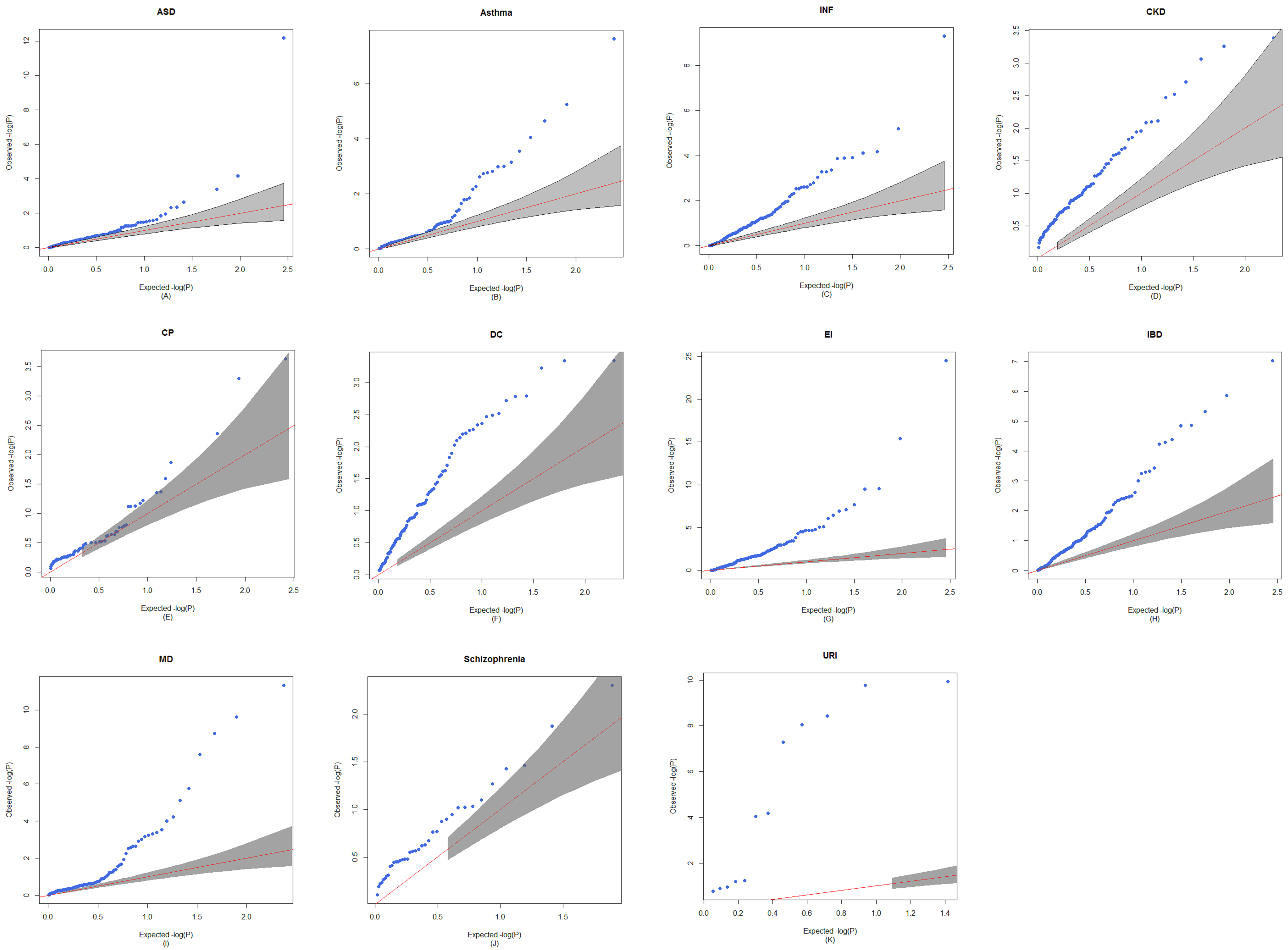

Figure S2: Quantile-quantile plots comparing p-value distributions of KEGG pathways across each of ASD and its co-morbidities with the theoretical quantiles. The plots are in $\log$-scale. (A) ASD, (B) Asthma, (C) Bacterial and viral infection, (D) Chronic kidney disease, (E) Cerebral Palsy, (F) Dilated Cardiomyopathy, (G) Ear infection, (H) IBD, (I) Muscular Dystrophy, (J) Schizophrenia, and (K) Upper Respiratory Infection.

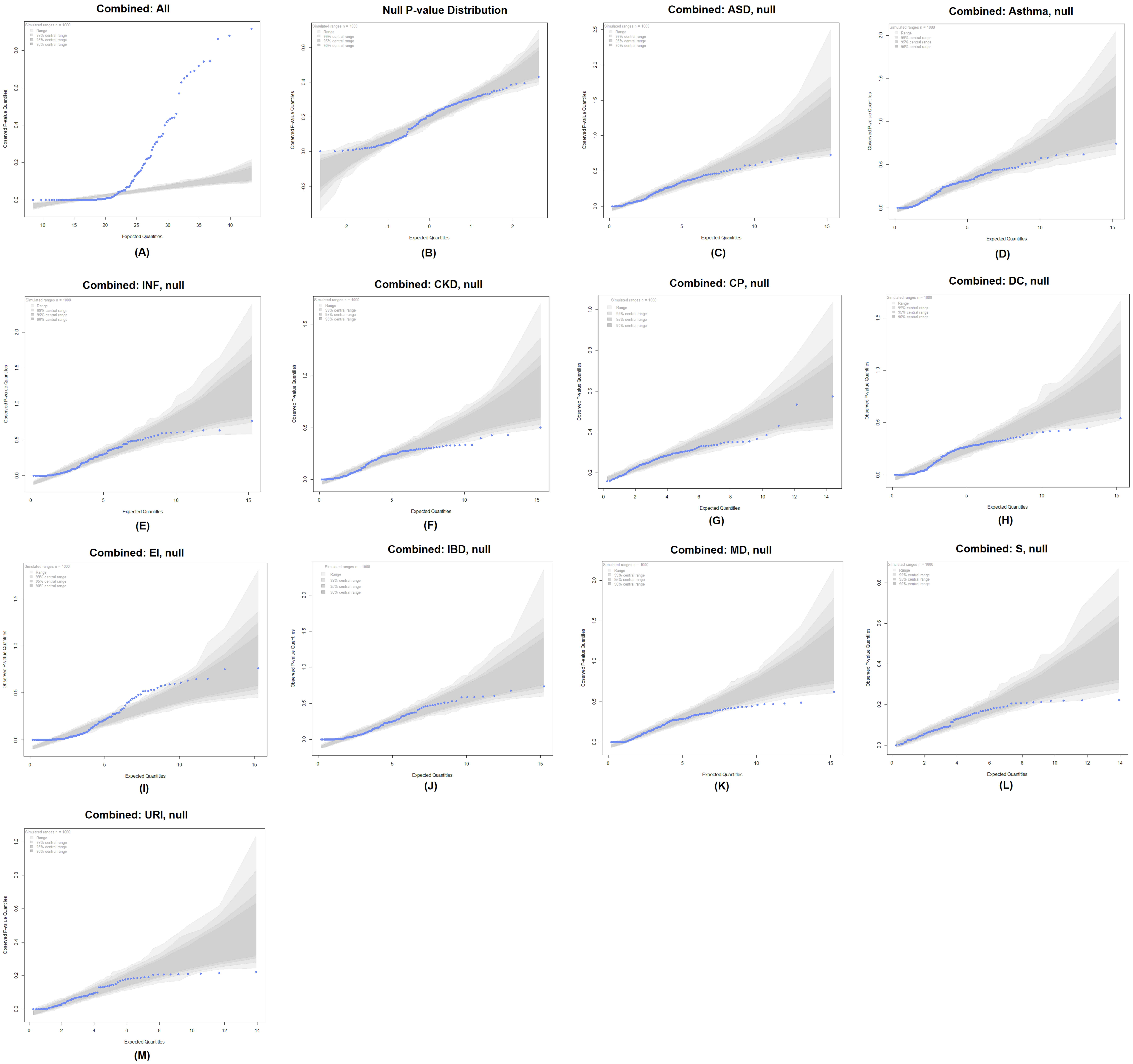

Figure S3: Quantile-quantile plots showing combined p-value distribution of KEGG pathways ASD and all its co-morbidities as well as combined p-value distribution of pathways for each disease with the null distribution. The combined p-values are compared with theoretical quantiles drawn from appropriate chi-square distributions and the null distribution is compared with theoretical quantiles from standard normal distribution.(A) All diseases combined, (B) Null distribution, (C) ASD and null, (D) Asthma and null, (E)Bacterial and viral infection and null, (F) Chronic kidney disease and null, (G) Cerebral palsy and null, (H) Dilated cardiomyopathy and null, (I) Ear infection and null, (J) IBD and null, (K) Muscular dystrophy and null, (L) Schizophrenia and null, and (M) Upper respiratory infection and null.

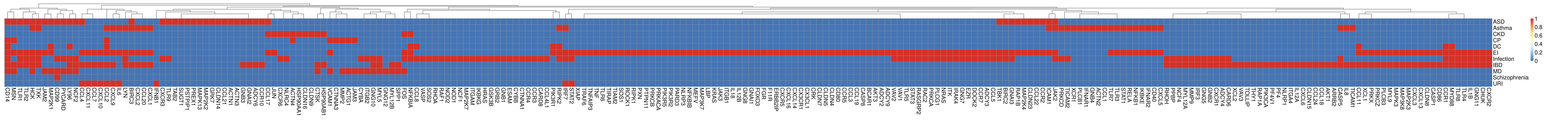

Figure S4: Binary heatmap showing the overlap between the disease gene sets and the union set of genes in the four significant innate immunity pathways from KEGG. A cell marked "Red" indicates an "overlap" whereas a cell marked "Blue" means "no overlap". For presentation purposes, the genes having no overlap with any of the diseases are omitted. Here, ASD = Autism Spectrum Disorder, CKD = Chronic Kidney Disease, CP = Cerebral Palsy, DC = Dilated Cardiomyopathy, EI = Ear Infection, IBD = Inflammatory Bowel Disease, Infection = Bacterial & Viral Infection, MD = Muscular Dystrophy, and URI = Upper Respiratory Infection.

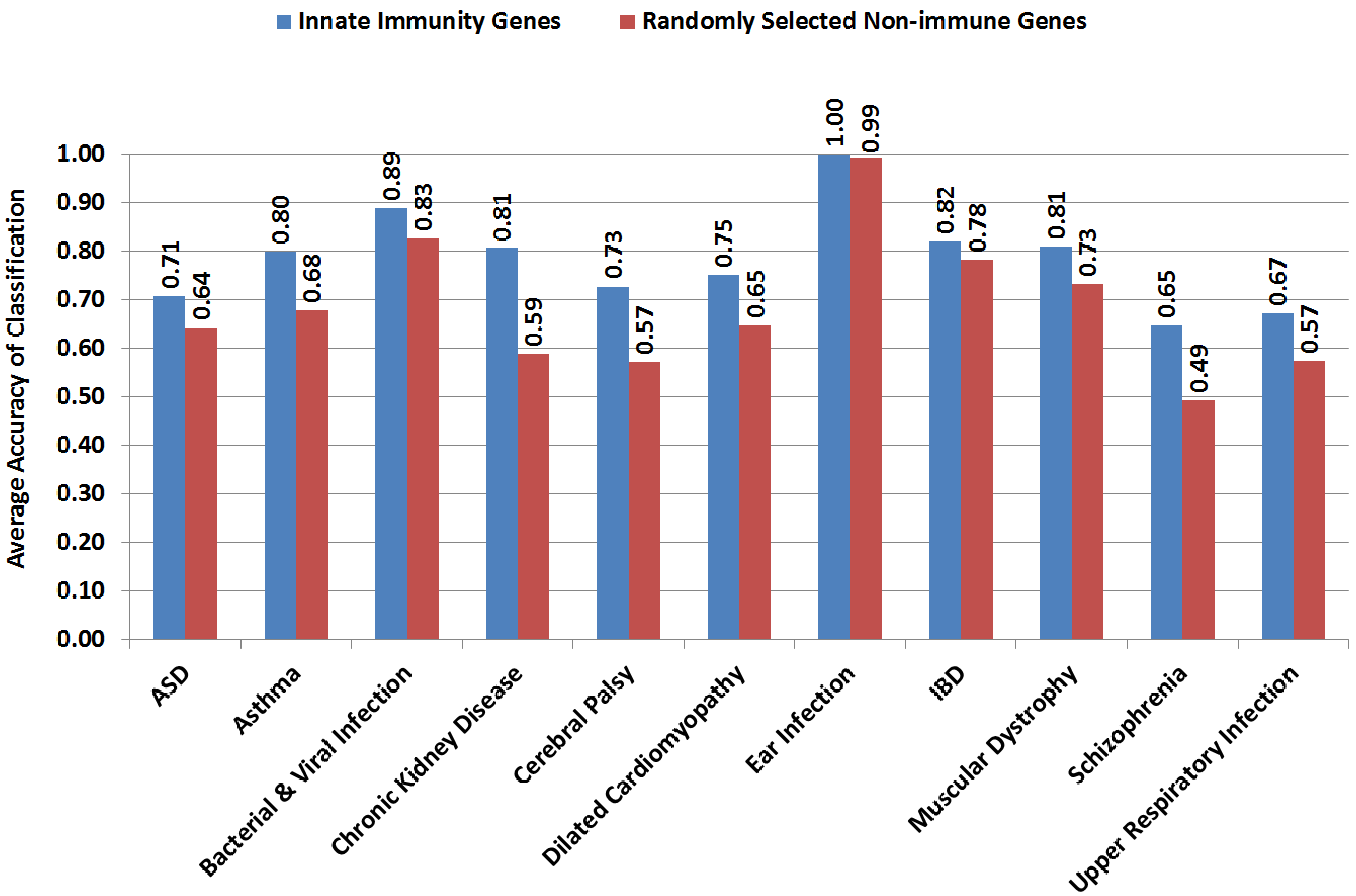

Figure S5: Accuracy of classification for case-control groups in different diseases using differentially expressed genes that overlap in the significant innate immunity related KEGG pathways versus randomly selected disease genes that do not overlap in the innate immunity pathways.
