# Supplementary material for: Integrative analysis of genetic data sets reveals a shared innate immune component in autism spectrum disorder and its co-morbidities
Source: Genome Biol. 2016 Nov 14;17:228. doi: 10.1186/s13059-016-1084-z (PMC5108086; doi:10.1186/s13059-016-1084-z)
Supplement: Additional file 7 — Supplementary text. This PDF file contains the supplementary text describing the four different classification techniques we used for classifying cases vs. controls in microarray gene expression data from ASD and its co-morbidities. (PDF 196 kb) [file 13059_2016_1084_MOESM7_ESM.pdf]

Supplementary Text for

"Integrative analysis of genetic datasets reveals a shared innate immune component in autism spectrum disorder and its co-morbidities"

Nazeen *et al.*

Different Classification Methods for Microarray Gene Expression Data

Different classification methods from the area of statistics and machine learning can be applied to microarray gene expression data of a disease, but there are some issues that make the task non-trivial. Gene expression data is very different from what is expected by these methods. First, it has very high dimensionality, usually contains tens of thousands of genes. Second, number of participating individuals in publicly available disease datasets is very small, often below 100. Third, most genes are irrelevant to disease case-control classification. Many researchers propose to do gene selection prior to classification which reduces the dimensionality as well as number of irrelevant genes. For disease datasets, one common practice is to consider the differentially expressed genes between cases and controls as predictors of the classifier.

There is no single classification method that is superior over the rest in terms of classifying disease gene expression data [1]. For such data, we want a binary classification method that gives maximal classification accuracy in distinguishing disease cases from controls. First we define the classification problem formally.

Problem Definition: Given a training set T = {(t_1, *case*), (t_2, *control*), … , (t_n, *case*)}, where n is the number of individuals in the training set, t_is are independent m-dimensional random data tuples of gene expression values, m is the total number of predictor genes, $t_i = (t_iX_1, t_iX_2, \dots, t_iX_m)$, m >> n and "*case*" and "*control*" are the class labels. Given a test set S = {s_1, s_2, … , s_l}. Each s_i is a gene expression data tuple of length m, and l is the number of individuals in the test set. Each s_i is in the form of $s_i = (s_iX_1, s_iX_2, \dots, s_iX_m)$, where X_j is the expression value of gene j. Find a classification function C, that gives maximal classification accuracy on S.

For completeness, we consider four classification methods namely, Fisher's Linear Discriminant Analysis (FLDA), K-Nearest Neighbor (KNN), Naïve Bayes Method (NB), and Support Vector Machine (SVM). For our purpose we found that SVM performed better than other classifiers in most of the cases in terms of accuracy.

Fisher's Linear Discriminant Analysis (FLDA):

FLDA tries to find a linear combination of the predictors that maximizes the separation between the centers of the data points from different classes while at the same time minimizing the variation within each class. This approach is often is preferred in practice due to its dimension-reduction property.

More formally, given a training set T and test set S as described above, FLDA tries to find the linear combination *Ma* of the columns of matrix M that maximizes the Rayleigh quotient given by a^TBa/a^TWa, where B is the between-class sum of squares, W is the within-class sum of squares, and a is the transformation matrix.

Let, μ_k be the vector of average gene expression values of m predictor genes for the training tuples in class k, where k ϵ {*case*, *control*}. The correlation between any test sample s_i and each class is measured using the squared Euclidean distance of s_i and μ_k, denoted by $d_k(s_i)$, where,

$$d_k(s_i) = \sum_{p=1}^{h} ((s_i - \mu_k)v_p)^2$$

Here, v_ps are linearly independent eigenvectors of the matrix $W^{-1}B$ and h is the number of its non-zero eigenvalues. Class k is assigned to s_i if the distance between s_i and μ_k is minimum. Thus, for training set T, and a test sample s_i, FLDA classifies s_i using the following classification function:

$$C(T, s_i) = argmin_k d_k(s_i)$$

FLDA was first proposed and implemented by R.A. Fisher in 1936 [2]. Since then it has been implemented numerous times for classifying gene expression data. We use the R implementation given by the *lda* function of the *MASS* package for our purpose.

K-Nearest Neighbor (KNN):

KNN is a distance metric based classifier. The main idea of this method is for each test sample s_i, find k training tuples from training set, T with most similar expression value according to a distance measure. The class label of s_i is assigned using majority vote from the selected k training tuples while breaking ties at random. Commonly used measure of similarities include, Pearson correlation, Euclidean distance etc. Thus, the classification function is given by,

$$C(T, s_i) = class\ of\ majority\ of\ k\ nearest\ neighbors\ of\ s_i\ in\ T$$

KNN was first proposed and implemented by Fix and Hodges [3] and was applied to gene expression data by Dudoit et al. for tumor classification [4]. We use the R implementation of *knn* function provided in the *class* package for our purpose. This implementation uses Euclidean distance as a measure of similarity. The value of k was chosen by iterating over values from 1 to 5 and picking the k which gives most accuracy in classification. In most cases the chosen value was either 2 or 3.

Naïve Bayes Method (NB):

NB method uses probabilistic induction to assign class labels to test samples, assuming independence among the predictor genes. The method models each class as a set of Gaussian distributions: one for each gene, by looking the gene expression values of the training samples. Let, G_k denote the class variable representing the setoff Gaussian distributions where k ϵ {*case*, *control*}. Then G_k is given by $G_k = \{ G_k^1, G_k^2, \ldots, G_k^m\}$ where, G_k^i is the Gaussian distribution of class k for gene i.

For training set T and any test sample s_i of test set S, the class label of s_i is obtained by the classification function:

$$C(T, s_i) = argmax_k^m \left(\sum_{g=1}^{m} \log P(s_i^g | G_k^g) \right)$$

$P(s_i^g | G_k^g)$ is given by Bayes rule and can be approximated from the mean and standard deviation of the Gaussian distribution for gene g from class k's distribution set, G_k^g.

NB method was first used to classify gene expression data in 2000 [5,6]. We use the R implementation of NB method given by *naiveBayes* function of the *e1071* package for our purpose.

Support Vector Machine (SVM):

SVM is a max-margin classifier that tries to find a hyperplane with maximum margin to separate the training tuples into different groups according to their classes. The margin of the hyperplane is defined as the distance from the hyperplane to the sets of points that are closest to it. The points that lie closest to the max-margin hyperplane are called *support* vectors. Since gene expression data can be viewed as very sparse points in a very high dimensional space, it is easy to find several hyperplanes that can separate the training tuples. But this method is often prone to overfitting.

Formally, let, T be the training set, as defined before, with n training samples of the form $x_i = (t_i, c_i)$ where, t_is are expression vectors of the form $(t_iX_1, t_iX_2, \ldots, t_iX_m)$ and $c_i \in \{1, -1\}$ is the class label with "1" representing cases and "-1" representing controls. Let, the max-margin hyperplane be denoted by the vector **w** and scalar b. Given a test sample s_i, SVM assigns class label to s_i based on the distance of s_i from the hyperplane in feature space. Thus the classification function is given by,

$$C(T, s_i) = \begin{cases} 1, if\ sign(\langle \boldsymbol{w}, \varphi(s_i)\rangle - b) > 0 \\ -1, otherwise \end{cases}$$

Here $\varphi(s_i)$ denotes the mapping of test sample s_i into the feature space and $\langle \boldsymbol{x}, \boldsymbol{y} \rangle$ denotes the dot product of two vectors **x** and **y**. SVM determines the max margin hyperplane by applying various dot product functions as kernels depending on the separability of the training data points.

SVM was first introduced by Vapnik et al. and used in many data mining applications [7-9]. We use the R implementation of SVM givevn by the *ksvm* function of the *kernlab* package for our purpose. We have applied both *vanilladot* (linear) and *rbfdot* (Gaussian) kernels and got similar levels of accuracy.

Aggregated classifiers are often useful for improving accuracy of classification. But since the main focus of our study is not getting a higher accuracy in classifying cases and controls of a disease dataset but to select the appropriate multiple hypothesis correction test which can give more informative genes, we limited our discussion to the basic classifiers.

References:

1. Lu, Y., & Han, J. (2003). Cancer classification using gene expression data. *Information Systems*, 28(4), 243-268.
2. Fisher, R. A. (1936). The use of multiple measurements in taxonomic problems. *Annals of Eugenics*, 7(2), 179-188.
3. Fix, E., & Hodges, J. L. (1989). Discriminatory analysis. Nonparametric discrimination: consistency properties. *International Statistical Review/Revue Internationale de Statistique*, 57(3), 238-247.
4. Dudoit, S., Fridlyand, J., & Speed, T. P. (2002). Comparison of discrimination methods for the classification of tumors using gene expression data. *Journal of the American Statistical Association*, 97(457), 77-87.
5. Keller, A. D., Schummer, M., Hood, L., & Ruzzo, W. L. (2000). Bayesian classification of DNA array expression data. Univ. Washington, Seattle, WA, USA, Tech. Rep. UW-CSE-2000-08-01.
6. Friedman, N., Linial, M., Nachman, I., & Pe'er, D. (2000). Using Bayesian networks to analyze expression data. *Journal of Computational Biology*, 7(3-4), 601-620.
7. Boser, B. E., Guyon, I. M., & Vapnik, V. N. (1992). A training algorithm for optimal margin classifiers. In Proceedings of the fifth annual workshop on Computational learning theory (pp. 144-152). ACM.
8. Burges, C. J. (1998). A tutorial on support vector machines for pattern recognition. *Data Mining and Knowledge Discovery*, 2(2), 121-167.
9. Vapnik, V. N., & Vapnik, V. (1998). Statistical Learning Theory (Vol. 1). New York: Wiley.
